# Age-related environmental gradients influence invertebrate distribution in the Prince Charles Mountains, East Antarctica

**DOI:** 10.1098/rsos.160296

**Published:** 2016-12-14

**Authors:** Paul Czechowski, Duanne White, Laurence Clarke, Alan McKay, Alan Cooper, Mark I. Stevens

**Affiliations:** 1 Australian Centre for Ancient DNA, University of Adelaide, Adelaide, South Australia 5005, Australia; 2 Antarctic Biological Research Initiative, Bolivar, South Australia 5110, Australia; 3 Institute for Applied Ecology, University of Canberra, Canberra, Australian Capital Territory 2601, Australia; 4 Australian Antarctic Division, Kingston, Tasmania 7050, Australia; 5 Antarctic Climate and Ecosystems Cooperative Research Centre, University of Tasmania, Hobart, Tasmania 7001, Australia; 6 Plant and Soil Health, South Australian Research and Development Institute, Waite Campus, Urrbrae, South Australia 5064, Australia; 7 South Australian Museum, Science Centre, Adelaide, South Australia 5000, Australia; 8 School of Pharmacy and Medical Sciences, University of South Australia, Adelaide, South Australia 5001, Australia

**Keywords:** Antarctica, invertebrates, environmental DNA, gradient, salinity, high-throughput sequencing

## Abstract

The potential impact of environmental change on terrestrial Antarctic ecosystems can be explored by inspecting biodiversity patterns across large-scale gradients. Unfortunately, morphology-based surveys of Antarctic invertebrates are time-consuming and limited by the cryptic nature of many taxa. We used biodiversity information derived from high-throughput sequencing (HTS) to elucidate the relationship between soil properties and invertebrate biodiversity in the Prince Charles Mountains, East Antarctica. Across 136 analysed soil samples collected from Mount Menzies, Mawson Escarpment and Lake Terrasovoje, we found invertebrate distribution in the Prince Charles Mountains significantly influenced by soil salinity and/or sulfur content. Phyla Tardigrada and Arachnida occurred predominantly in low-salinity substrates with abundant nutrients, whereas Bdelloidea (Rotifera) and Chromadorea (Nematoda) were more common in highly saline substrates. A significant correlation between invertebrate occurrence, soil salinity and time since deglaciation indicates that terrain age indirectly influences Antarctic terrestrial biodiversity, with more recently deglaciated areas supporting greater diversity. Our study demonstrates the value of HTS metabarcoding to investigate environmental constraints on inconspicuous soil biodiversity across large spatial scales.

## 1. Introduction

There is an urgent need for information about Antarctic terrestrial biodiversity and the relationship with environmental constraints in order to predict the effects of anticipated human-mediated environmental change on Antarctic biota [1] and for successful conservation management [2,3]. A valuable approach for exploring potential impacts of environmental change on ecosystems is to compare biodiversity patterns across environmental gradients [4]. For example, comparing ecosystems over latitudinal or altitudinal gradients can allow predictions of biodiversity changes in response to increasing temperature [5], but limited numbers of sites and samples may constrain predictive power [6]. Consequently, baseline data for predicting future environmental changes across Antarctica need to describe biodiversity across large spatial scales in relation to as many environmental variables as possible [7,8].

It has previously been suggested that the large-scale distributions of most Antarctic terrestrial fauna are determined by geo-glaciological events and the presence of past refugia rather than latitudinal variations in climatic and environmental conditions [9]. On smaller spatial scales, Antarctic invertebrate biodiversity is associated with low salinity and high nutrient content [10–13], with the exception of some nematode [14] and rotifer species [6]. Typically, a set of interrelated soil and environmental factors determines the abundance and composition of Antarctic soil communities [15] and hence multivariate statistics are well suited to study such relationships [9]. A multivariate statistical approach linking many environmental variables to all major Antarctic invertebrates could be used to elucidate whether the broader distribution of Antarctic invertebrate taxa is strongly influenced by past geo-glaciological events or rather is correlated with environmental constraints as observed at small spatial scales.

Amplicon sequencing (metabarcoding *sensu lato*) supported by high-throughput sequencing (HTS) technology is a promising approach to rapidly obtain biodiversity information from a large number of samples in extreme environments such as Antarctica [17]. For Antarctic invertebrates, morphological approaches require a high level of taxon-specific knowledge and are logistically constrained to small sample numbers [18–21]. Additionally, morphologically cryptic Antarctic species may consist of multiple genetic lineages shaped by long-term isolation, making molecular approaches a more suitable tool to investigate their diversity [11,22,23]. HTS metabarcoding approaches have now been used to describe invertebrate distribution and diversity on a global scale, excluding the Antarctic region [24]. These methods could also provide valuable information regarding the environmental determinants of Antarctic invertebrate biodiversity [25].

In a previous study [26], we used HTS of 18S ribosomal DNA (18S rDNA) to describe eukaryotic diversity from 12 sites in the Prince Charles Mountains (PCMs), East Antarctica, revealing trends in diversity related to latitudinal and elevational gradients. In this study, we provide a detailed analysis of the environmental determinants for the four predominant Antarctic invertebrate phyla (nematodes, rotifers, tardigrades and arthropods) across a much larger spatial scale in the PCMs using multivariate statistics. To do so, we initially characterized the spatial variation of soil geochemical and mineral composition in the PCMs. By combining this environmental predictor data with HTS information of 18S rDNA, we show that the distribution of invertebrates in the PCMs is strongly influenced by salinity measured as electrical conductivity and sulfur content, which are themselves correlated with terrain age. These findings suggest that long-term soil formation processes unique to Antarctica are driving invertebrate distribution across large spatial scales.

## 2. Material and methods

### 2.1. Fieldwork

**Figure 1.**
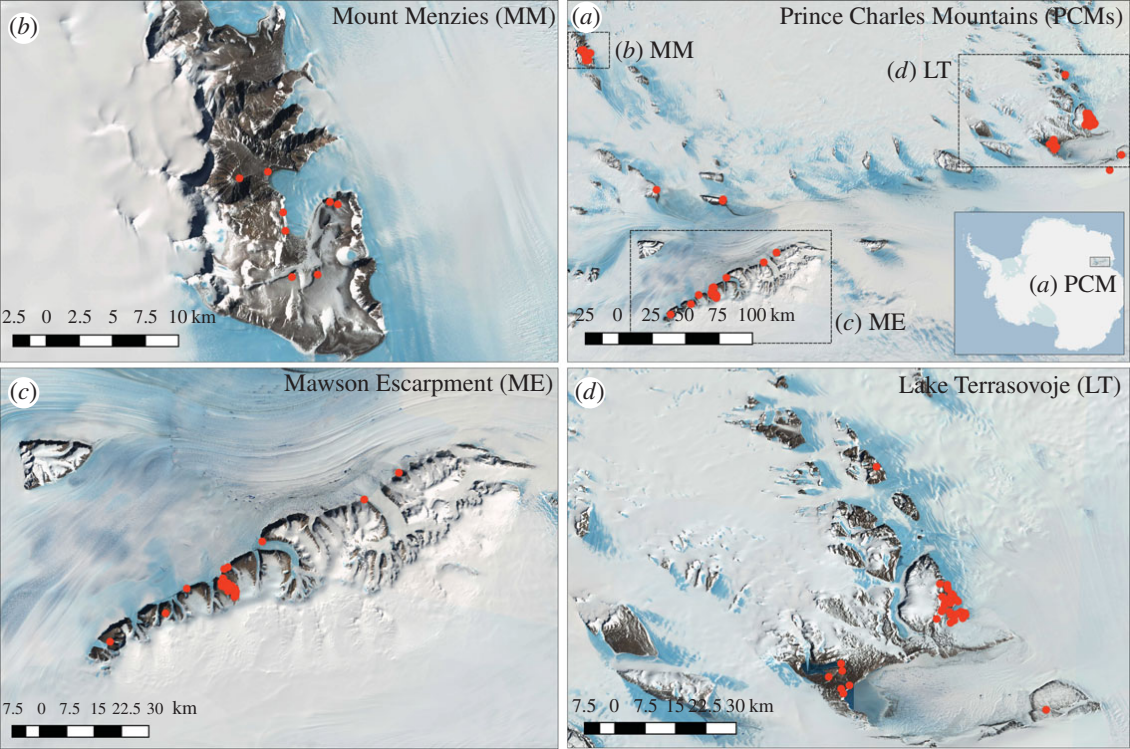
Sampling locations yielding invertebrate phylotypes in the Prince Charles Mountains, East Antarctica. (*a*) Prince Charles Mountains (PCMs) with sampling locations in overview, with (*b*) Mount Menzies (MM), (*c*) Mawson Escarpment (ME, including two locations north of the magnified area) and (*d*) Lake Terrasovoje (LT). Of 136 samples analysed in this study, 90 samples yielded invertebrate DNA, with eight (of 20) samples from MM, 38 (of 64) from ME and 44 (of 52) samples from LT. Median elevation for invertebrate observations was 1828 m.a.s.l. (MM), 995 m.a.s.l. (ME) and 149 m.a.s.l. (LT). CIRREF Imagery courtesy of the US Geological Survey, distributed with Quantarctica. Quantarctica package courtesy of the Norwegian Polar Institute, visit www.quantarctica.org.

Fieldwork was conducted in the PCMs (East Antarctica; figure 1*a*) from 26 November 2011 to 21 January 2012 at Mount Menzies (MM), Mawson Escarpment (ME) and Lake Terrasovoje (LT), and is described in detail elsewhere [26]. A total of 136 samples were considered for this study, with 20 from MM, 64 from ME and 52 from LT.

### 2.2. Soil geochemical and mineral measurements

Soil geochemical analysis was performed at the CSBP Soil and Plant Analysis Laboratory (Bibra Lake, AU-QLD) with standardized analysis methods [27] as listed in the electronic supplementary material, table S1). Across all samples, we considered the continuous metric soil geochemical variables $\text{N}{{\text{H}}_{4}}^{+}$, $\text{N}{{\text{O}}_{3}}^{-}$, K, P, S, pH (for CaCl_2_ and H_2_O), organic C content and conductivity as a measure of substrate salinity [12] . Compositional measures of mineral abundances across all samples included the minerals quartz, feldspar, titanite, pyroxene/amphibole/garnet, micas, dolomite and kaolin/chlorite, which were derived from X-ray diffraction spectra. To collect these spectra, soil subsamples were initially dried at 100°C for 48 h and then sieved through 2 mm and 63 µm meshes. The resulting powders (less than 63 µm) were analysed using a BTX II Benchtop XRD®, (Cu-K*α* X-ray source), with 105 consecutive cycles per sample. Mineral identification was conducted using PANalytical's Highscore Plus software v. 3.0e, against the open crystallographic database [28]. Mineral groups were considered present if position and intensity of phase-identified peaks matched three or more peaks in the database as described elsewhere [29].

### 2.3. Preparation of environmental predictor observations

Analyses of geochemical and mineral predictor data were conducted in R v. 3.3.1 [30]. Among 792 soil geochemical predictor measurements, initially nine values with the largest difference from the mean were replaced by their means, to mitigate detrimental effect on principal component analysis (PCA) [31]. To subsequently meet assumptions of normality and variance uniformity across environmental predictors, the mineral compositional data were log-ratio transformed with R package Rgr 1.1.13 [32] in order to remove the closure effect of compositional data [33]. Combined mineral composition and soil geochemical variables were then Yeo-Johnson transformed [34], scaled to unit variance and centred, using R package Caret v. 6.0-71 [35]. Lastly, highly (more than 0.75) correlated predictor variables were removed based on the Pearson correlation coefficients [31], using R's Stats package [30] (conductivity correlated with S, pH CaCl_2_ correlated with pH H_2_O and titanite correlated with feldspar, the latter of each pair retained). Geomorphic mapping and weathering studies [36,37] and cosmogenic exposure dating [38] were used for age determination of glacial sediment; mean values across a lower and higher estimate were used for regression analysis. The documented code for this analysis is supplied in the electronic supplementary material, main analysis.

### 2.4. Preparation of biological response observations

Preparation and analyses of biological response data were conducted in QIIME v. 1.9.0 [39] and R v. 3.3.1 [30] after laboratory work, based on considerations outlined in our earlier review [40]. Detailed methods and materials for molecular laboratory work (DNA extractions, library generation and sequencing) and retrieval of invertebrate phylotypes (equivalent to Molecular Operational Taxonomic Units *sensu lato* [41,42]) are provided in the electronic supplementary material, text. In essence, DNA extractions and PCR amplification of 18S rDNA were conducted as per Czechowski *et al.* [26], with the exception of using three instead of two PCR replicates. To amplify a 125 bp region of the eukaryotic 18S rDNA, the primers ‘Euk1391f’ and ‘EukBr’ [43] were used. During initial tests, those primers successfully amplified rotifers, tardigrades, nematodes and arthropods (Arachnida) in bulk positive controls (electronic supplementary material, text and figure S3). Following sequencing, phylotype data were pre-processed using QIIME. After filtering the raw sequence data based on quality scores (*Phred* score 19), and removal of chimeras in a de novo approach employing Usearch v. 6.1. [44], phylotype clustering was performed as described in Czechowski *et al.* [26] and taxonomy assigned using the Silva database v. 119 [45]. To allow abundance correction using the CSS algorithm [46] instead of depreciated resampling approaches [47], stringent filtering removed all phylotypes covered by less than 100 sequences and all samples with less than 1000 reads, analogous to, but more stringent than, other metabarcoding studies [48,49]. All taxonomic assignments to eukaryotic sequence data were verified using current taxon-specific literature. QIIME-generated phylotype tables were then further handled in R, using the package Phyloseq v. 1.16.2 [50]. Filtering the biological data for invertebrates resulted in sparse observations and concomitant high site heterogeneity, initially preventing generation of meaningful distance matrices for ordination approaches. Phylotypes were thus agglomerated on the class level. The documented code for this analysis is supplied in the electronic supplementary material, analysis scripts.

### 2.5. Ordination of environmental predictors and biological responses

Environmental predictors were related to biological responses (i.e. invertebrate phylotypes) by means of non-metric multidimensional scaling (NMDS) [51,52] as implemented in R package Vegan v. 2.4-1 [53,54]. Initially, the biological space was defined using Bray dissimilarities [55] between Wisconsin double standardized, square root transformed sample-specific abundances. Environmental predictors were fitted to this ordination space using Vegan's ‘envfit()’ function and 9999 permutations. Significant environmental vector fits were subsequently tested using distance-based permutational multivariate analysis of variance (PERMANOVA, implemented in Vegan function ‘adonis()’) [56] and canonical correspondence analysis (CCA) [57]. In PERMANOVA, we tested the hypothesis of distance-expressed invertebrate class β-diversity being dependent on means of substrate S content. During CCA, we tested the hypothesis of substrate S contents representing a major gradient in the biological dataset. CCA evaluation was performed using Vegan-specific ANOVA functions (all with 999 permutations), including testing the significance of the ordination, its axis, variable addition (Type I test) and variable elimination (Type III test). Furthermore, variance inflation factors (VIFs) were used to assess variable significance, and the goodness of fit for each invertebrate class was retrieved. The documented code for this analysis is supplied in the electronic supplementary material, main analysis.

## 3. Results

### 3.1. Environmental data

**Figure 2.**
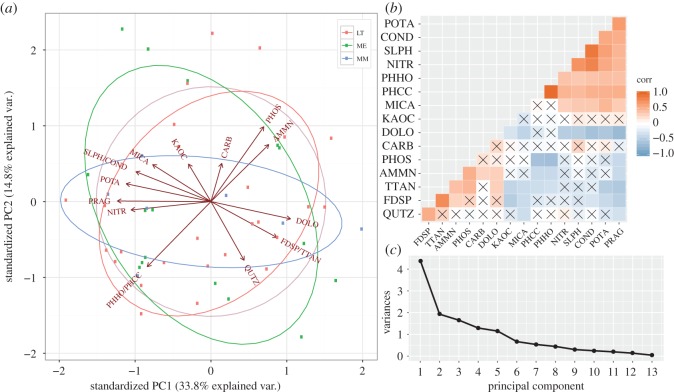
Analysis summary of environmental predictors. (*a*) Principal component analysis (PCA), (*b*) removal of correlated variables and (*c*) eigenvector variance for each principal component. (*b*) Highly (more than 0.75) correlated predictor variables were removed based on Pearson correlation coefficients, conductivity was correlated with S, pH CaCl_2_ correlated with pH H_2_O and titanite correlated with feldspar, the former of each pair was removed. (*a*) As summarized by PCA, all regions shared many characteristics across environmental predictors. Coloured circles indicate normal range of principal components (PCs) for each location, central faint circle for comparison purposes. (*c*) Owing to the heterogeneity of the sampling locations, eigenvector variance declined gradually.

Yeo-Johnson transformation, scaling and centring of environmental predictors considerably improved normality and variance uniformity, with some variables ($\text{N}{{\text{H}}_{4}}^{+}$, $\text{N}{{\text{O}}_{3}}^{-}$, K, P and C) exhibiting bimodal distributions due to measurements below the detection limit ($\text{N}{{\text{H}}_{4}}^{+}$and P at MM), or heteroscedasticity across measurement values ($\text{N}{{\text{O}}_{3}}^{-}$, K, and C; electronic supplementary material, main analysis, figures S8 and S9). While three highly positively correlated variables were removed (see methods in §2.3), most other correlations were below the selected threshold and insignificant (figure 2*b*). Compared with the mean measurement of both other regions, MM soils were typically richer in $\text{N}{{\text{O}}_{3}}^{-}$, slightly alkaline, and rich in quartz, micas and kaolin/chlorite. Soils of ME were overall rich in K, S and C, with above-average values of pyroxene/amphibole/garnet and dolomite. Lake Terrasovoje was comparatively rich in $\text{N}{{\text{H}}_{4}}^{+}$ and P, and dominated by feldspar (electronic supplementary material, main analysis, figure S9). The heterogeneity of substrates impaired the establishment of terrain characteristics across the three sites (electronic supplementary material, main analysis, figures S8 and S9), with all three regions sharing many characteristics across environmental predictors, as summarized by PCA (figure 2*a*). Consequently, PCA variance decline across principal components was shallow (figure 2*c*). Soil salt concentrations (expressed through variables conductivity, S, $\text{N}{{\text{O}}_{3}}^{-}$ and P; electronic supplementary material, main analysis, pages 30*ff*) across the sampling area consistently increased with estimated mean sample substrate ages, and these age–salt regressions were most significant for $\text{N}{{\text{O}}_{3}}^{-}$ (*ad. R^2^* = 59%, *p *= 1.68 × 10^−7^), S (*ad. R^2^* = 46%, *p *= 9.078 × 10^−6^) and conductivity (*ad. R^2^* = 59%, *p *= 1.68 × 10^−7^). This trend was most pronounced at MM for conductivity, S and $\text{N}{{\text{O}}_{3}}^{-}$, with *R*^2^ values of 99%. At ME, age–salt regressions had *R*^2^ values of 17 to 26%. At the more coastal LT, correlations ranged from 51 to 83%. In combination, conductivity means across the three locations and trends described by regression analyses were indicative of higher salt accumulation rates in drier inland areas (MM, ME), when compared with a more coastal site (LT), where salts may be more frequently flushed out of the substrata by glacial meltwater.

**Figure 3.**
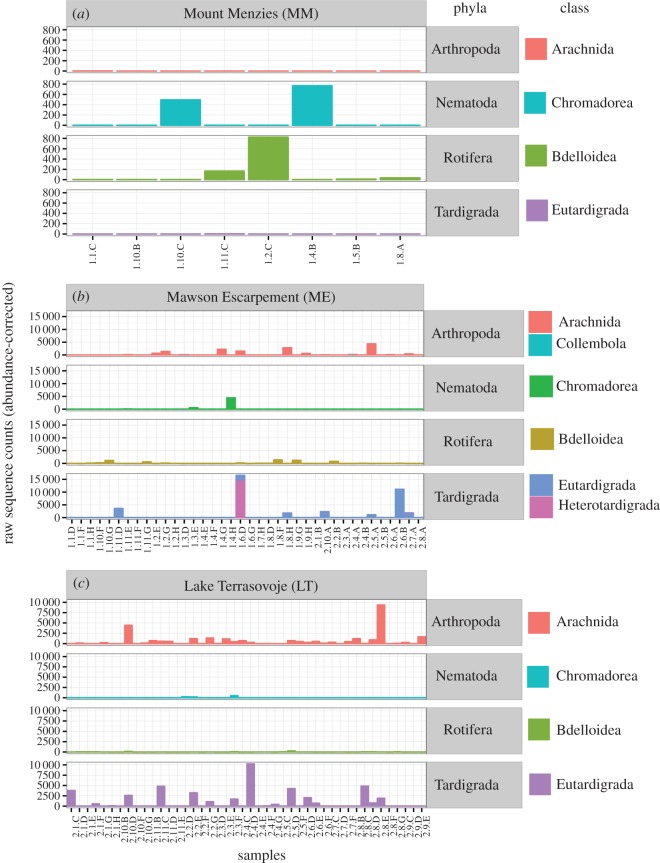
Raw sequence counts of invertebrate phyla and classes recovered from (*a*) Mount Menzies (MM), (*b*) Mawson Escarpment (ME) and (*c*) Lake Terrasovoje (LT). Note that non-abundance-corrected counts were used for this graphic. (*a*,*c*) Class–phylum relationships are bijective as indicated by colours. (*b*) At ME two classes (Eutardigrada and Heterotardigrada) were detected for phylum tardigrade and for phylum Arthropoda (Arachnida and Collembola). Very low sequence counts may not be visible for some taxa (e.g. phylum Arthropoda, class Collembola, ME).

**Figure 4.**
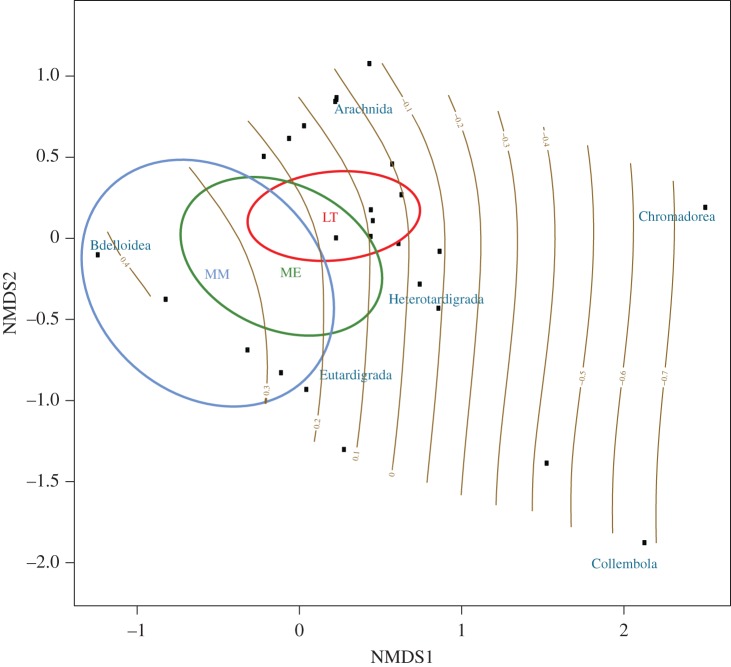
Non-metric multidimensional scaling of invertebrate classes (blue labels). Invertebrate composition determines location if sampling sites in biological space (black squares). For sampling locations, 95% confidence intervals are indicated for MM (blue), ME (green) and LT (red), and approximate the geographical setting (figure 1). The only significant environmental gradient that could be fitted to the ordination is described by S and correlated conductivity measurements (yellow isoclines). This gradient runs from high (MM), over ME to its lowest values (LT) corresponding to a decline in salinity from south to north (figure 1).

Nematodes can be found widely distributed in the McMurdo Dry Valleys [14,58], but in our study were predominant in the harshest environment (MM, figure 3*a*), in conjunction with rotifers, with an unpronounced link to high-salinity substrates (figure 4). Nematode species of the class Chromadorea may be affected differently by environmental constraints. For instance, the abundance of *Scottnema lindsayae* was negatively correlated with soil moisture and C content, [6,14] while *Plectus antarcticus* was dominant in wet soils with low salinity [6]. The dominance of Chromadorea in high-salinity areas (figure 3) is conceivable due to species-specific physiological traits [66], allowing species such as *S. lindsayae* to be almost ubiquitously distributed [10] if not affected by predation, which is more likely in moist and nutrient-rich areas [58]. Additionally, in overall highly saline areas (MM) Nemetoda would still be more likely to be found in substrates with locally minimal salt concentrations [12].

### 3.2. Biological data

Of 136 samples analysed in this study, 90 samples (figure 1) yielded invertebrate DNA (66%), with eight (of 20) samples from MM (40%), 38 (of 64) from ME (59%) and 44 (of 52) samples from LT (84%). Median elevation for invertebrate observations was 1828 m.a.s.l. (MM), 995 m.a.s.l. (ME) and 149 m.a.s.l. (LT). In absolute, non-abundance-corrected sequence counts (figure 3), bdelloid rotifers and Chromadorea (Nematoda) were dominant at MM, with tardigrades and arthropods (Arachnida) lacking (figure 3*a*). Absolute read counts for arthropods, nematodes, rotifers and tardigrades were comparatively higher at ME, with location-specific read counts highest for tardigrades and arthropods (figure 3*b*). LT was dominated by arthropods and tardigrades (figure 3*c*).

### 3.3. Biological data in relation to environment

NMDS (stress = 0.044, *R^2^* = 99.8% for non-metric fit and *R^2^* = 99.3% for linear fit) dispersed invertebrate classes widely across the biological space, indicative of scarce observations for all taxa (figure 4), but with 95% confidence intervals of all three locations resembling the geographical setting of the sampling area (compare figures 1 and 4). Of all 13 predictors, S with *R^2^* of 16.7% (*p =* 0.126, correlated with variable conductivity) was the only environmental variable that could be fitted to the biological space in a meaningful way. Consistent with results described above, bdelloid rotifers were more likely to be observed in highly saline areas (mostly MM), while all other invertebrates occurred more frequently at locations with decreasing S content (i.e. at ME and LT). These results were corroborated by PERMANOVA (*R^2^* = 83%, *p* = 0.0074) and CCA (marginal tests for axes: *p =* 0.033, sequentially added terms: *p =* 0.042, marginal effect of terms *p =* 0.035, VIF = 1, and goodness of fit: Bdelloidea > Chromadorea > Collembola > Arachnida > Eutardigrada > Heterotardigrada).

## 4. Discussion

### 4.1. Salinity and nutrient availability are major determinants of invertebrate distribution

Our results indicate that soil salinity, expressed through conductivity, and/or correlated variable S, is the most important constraint on invertebrate biodiversity in the PCMs along a gradient which resembles the geographical setting of the sampling area (figure 4). Studies of the McMurdo Dry Valley soils (Antarctica) previously suggested that salinity is an important factor influencing diversity of nematode communities [10,14,58]. A similar effect was also observed for mites and other invertebrates in temperate latitudes [59–61]. *Cold desert* soils in this study (MM, ME) are dominated by the age-related accumulation of soluble salts from atmospheric deposition and weathering due to lack of available water [13,62]. In such soils, more complex communities were associated with younger, weakly developed drifts with low salinity [12]. As shown here for invertebrates and in Czechowski *et al.* [26] for all eukaryotes, biodiversity is analogously low for MM, but higher for ME. The effect of age-related salt accumulation is less pronounced in *polar desert* soils [13,63], such as encountered here at LT. At the lower latitude LT, factors other than soil salinity, such as nutrient input, influence soil biodiversity as higher moisture availability promotes washout of salts and other chemicals from the substrata.

### 4.2. Halo-tolerance or nutrient availability determine access to feeding resources

Along an elevation gradient in Taylor Valley (McMurdo Dry Valleys) invertebrate biodiversity was greatest at the lowest elevation, where soil moisture, carbon and nitrogen were highest and salinity was lowest [10]. In coastal Victoria Land (Cape Hallett), variation in soil metazoan communities was related to differences in soil organic matter and moisture levels [6]. Although sparse data for Chromadorea (figure 3) may have impaired their placement in the ordination space (figure 4), our results correspond well with those previous studies identifying habitat preferences for Bdelloidea and Chromadorea. These taxa are found more often in highly saline areas (figure 3), predominantly encountered at MM and ME (figure 2*a*); arachnids and tardigrades, on the other hand, tend to be more abundant in patches of nutrient-rich locations, predominantly at ME and LT (figures 2*a*, 3 and 4). The observed habitat preferences for different invertebrate taxa are linked to substrate salinity, because salinity may affect soil biodiversity through constraining the amount of available food or by affecting physiological functions [10], such as freeze tolerance [64]. While most invertebrates are confined to low-saline substrates due to physiological constraints (here mostly LT), more halo-tolerant invertebrate taxa may be able to feed on halo-tolerant micro-eukaryotes in areas of high salinity [65].

Nematodes can be found widely distributed in the McMurdo Dry Valleys [14,58], but in our study were predominant in the harshest environment (MM, figure 3*a*), in conjunction with rotifers, with an unpronounced link to high-salinity substrates (figure 4). Nematode species of the class Chromadorea may be affected differently by environmental constraints. For instance, the abundance of *Scottnema lindsayae* was negatively correlated with soil moisture and C content, [6,14] while *Plectus antarcticus* was dominant in wet soils with low salinity [6]. The dominance of Chromadorea in high-salinity areas (figure 3) is conceivable due to species-specific physiological traits [66], allowing species such as *S. lindsayae* to be almost ubiquitously distributed [10] if not affected by predation, which is more likely in moist and nutrient-rich areas [58]. Additionally, in overall highly saline areas (MM) Nemetoda would still be more likely to be found in substrates with locally minimal salt concentrations [12].

In this study, arachnids and tardigrades were found more frequently associated with nutrient-rich soils at LT and ME (figures 3 and 4). Tardigrades were previously associated with higher soil moistures [14], ubiquitously in coastal and inland sites [6,67], and ornithogenic nutrient deposits [68], and the majority of Antarctic mite species have similar habitat preferences [69]. Ornithogenic deposits are a major nutrient source of Antarctic soils, but at our sampling sites were only observed at ME and LT. Consequently, nutrients are more abundant at coastal sites [62].

### 4.3. Technical considerations

Linking biodiversity to environmental gradients is difficult in Antarctica. Various interrelated soil factors such as soil moisture, salinity and pH may modify the effects of soil carbon and nitrogen on biodiversity, and all variables collectively define suitable or inhospitable habitats, both at local and more regional levels [10,11,14,15]. As a consequence, several studies have not been able to find a clear link between environmental variables and invertebrate distribution, probably because terrestrial Antarctica is characterized by limited ice-free ground and a high degree of soil heterogeneity across varying spatial scales [7]. Interpreting explanatory variables with regard to biodiversity distribution may also be complicated by effects of spatial autocorrelation [9]. Considering the properties of the Antarctic terrain, our study delivers compelling insight into the relationships between biodiversity and constraints of its distribution imposed by highly heterogeneous environmental factors.

Slope and elevation were not considered in this study. Our previous work [26] revealed broad biogeographic trends related to altitude and latitude, while the underlying mechanisms evoking such trends remained indistinct. In line with this, it has previously been suggested that spatial variables may constitute surrogates for relevant environmental variables at Cape Hallett [70]. Elevation was found to covary with soil properties such as carbon, nitrogen and salinity at Taylor Valley [10]. In the Antarctic, elevation may be a strong proxy for salinity, as increasing elevation and lower temperature can increase the osmotic concentration of the soil, inhibiting biological activity [10,14,71]. Furthermore, elevation has been shown to be a proxy for soil temperature, melt availability and active layer depth in the region surrounding the PCMs [37]. Slope has been suggested to influence Arctic soil biodiversity due to the increased moisture run-off [68]; however, such an effect was deemed dependent on the moisture retention capabilities of local soils [70]. Hence, a relationship between slope angle and moisture should not be generalized without taking into account other variables, such as salinity. Regardless, it is possible that slope and elevation may influence Antarctic invertebrate distribution at smaller spatial scales or at finer taxonomic resolution than examined here.

Annual mean temperature and water availability were not considered in this study. Owing to practical constraints, we were unable to obtain representative time series data from the remote Antarctic locations addressed in this work. Spot measurements of temperature and water availability are a poor proxy for biologically relevant long-term values at a given sampling site [70,72]. Several possible modelling approaches to include such time series data are reviewed elsewhere [8], and should be applied once such data are available.

Each site was sampled only once in our study. Consequently, we cannot rule out the possibility of seasonal variation in the invertebrate communities at these locations. However, we sampled during the austral summer when biological activity is highest, and consequently our results would reflect the most ecologically significant invertebrate community. Also, the relatively large amount of soil processed, the short markers used (125 bp) and high potential for DNA preservation in polar environments [73] would allow the detection of environmental DNA. Hence, the detected communities are effectively a composite sample integrated over time, averaging out seasonal variation to some degree. For these reasons, we feel our conclusions would not be altered by variation in the invertebrate community over time.

### 4.4. Soil salinity as a threat to global soil quality

Soil organisms are critical to maintain soil quality in most ecosystems [74], but human-mediated soil salinization threatens this biodiversity [75–77]. Antarctic soil ecosystems are relatively simple and provide a system to explore the effect of abiotic factors while mostly lacking influence of confounding complex biotic interactions. Our results demonstrate major changes in soil invertebrate communities over salinity gradients, with entire taxonomic classes being absent in highly saline areas (figures 3 and 4). Increasing salinization of arable soils [76] is likely to have major impacts on soil invertebrate communities and ecosystem function [59,60,78]. With anticipated sea-level rise and shortages of fresh water, deterioration of arable soils by salinization may be further exacerbated [60]. Consequently, our work contributes towards understanding the effect of soil salinization on soil communities in northerly latitudes. Furthermore, our standardized approach can serve as an example for soil quality monitoring across large spatial scales in difficult terrain [75].

### 4.5. Conclusion

It has previously been suggested that geo-glaciological events and the presence of glacial refuges may be more important than latitudinal variations in climatic and environmental conditions in determining the large-scale distributions of most Antarctic terrestrial fauna [9]. Indeed, areas of Antarctica recently deglaciated and/or receiving meltwater, such as around Lake Terrasovoje [38], are likely to represent diversity hotspots for invertebrates (as shown here) and potentially other eukaryotes [26], and thus warrant further research into their conservation value [3]. Additionally, we corroborate that the unique properties of Antarctic soils strongly constrain the distribution of Antarctic terrestrial taxa owing to long-term salt accumulation in long-exposed dry inland areas, due to more complex patterns [13] than explainable with the deglaciation during the last glacial maximum [38]. Consequently, the impact of geo-glaciological events and the presence of glacial refuges should be analysed in combination with environmental parameters to have a better understanding of Antarctic biogeographic patterns.

## Supplementary Material

ESM contains supplemental methods and material as well as rendered source code of the main analysis (incl. figures). All other information is linked to this manuscript via GitHub and Zenodo (https://github.com/macrobiotus/antarctic_invertebrates.git)

## Data accessibility

Supplemental methodological information and results are available in the electronic supplementary material. The documented code to conduct analyses is maintained at https://github.com/macrobiotus/antarctic_invertebrates.git, the release used here is available via http://dx.doi.org/10.5281/zenodo.190926. R objects and sequence information used here are available via http://dx.doi.org/10.5281/zenodo.162484.
