# Supplementary material for: Age-related environmental gradients influence invertebrate distribution in the Prince Charles Mountains, East Antarctica
Source: R Soc Open Sci. 2016 Dec 14;3(12):160296. doi: 10.1098/rsos.160296 (PMC5210674; doi:10.1098/rsos.160296)
Supplement: ESM contains supplemental methods and material as well as rendered source code of the main analysis (incl. figures). All other information is linked to this manuscript via GitHub and Zenodo (https://github.com/macrobiotus/antarctic_invertebrates.git) [file rsos160296supp1.pdf]

Age-related environmental gradients influence invertebrate distribution in the Prince Charles Mountains, East Antarctica

Supplemental Materials

Paul Czechowski, Duanne White, Laurence Clarke, Alan McKay, Alan Cooper, Mark I. Stevens

5th December 2016

Contents

1 Supplemental information **1**

1.1 Design of molecular work and control reactions . . . 1

1.2 Primer design . . . 1

1.3 Extraction, amplification and sequencing . . . 2

1.4 Deconvolution and chimera screening . . . 2

1.5 Reference data and phylotype definition . . . 2

1.6 Filtering and abundance correction . . . 2

2 Data and analysis scripts **2**

3 Supplemental figures **4**

4 Supplemental tables **8**

1 Supplemental information

1.1 Design of molecular work and control reactions

Antarctic soil DNA extracts (see section 1.3 for extraction details) from the Prince Charles Mountains were received in seven micro-titre plates, aliquots were then selected across two plates. Several wells of each plate were reserved for control reactions. Controls included aliquots of blank extracts, H_2O (prior to amplification) or blank PCRs (after amplification) and several positive controls (Fig. 1) including mock DNA mixtures with Australian insects [1], or whole-soil extracts from Australian soils. For further information please refer to our earlier work [2].

1.2 Primer design

The forward 18S primer sequence consisted of an Illumina adapter, a primer pad and linker, as well as the target-priming region "1391f" [3, 4]. The reverse primer sequence contained the reverse complement of a 3' Illumina adapter, a twelve bp recognition sequence allowing error correction [5] (assigned to samples as shown in Fig. 2, following naming of [4]), a reverse primer pad and linker, as well as the reverse primer "EukBr" [3, 4]. Specificity of primers for target taxa was tested with positive controls (Fig. 2). For further information please refer to our earlier work [2].

1.3 Extraction, amplification and sequencing

Extractions were performed using a method optimised for the retrieval of DNA from different soil types and invertebrates [6, 7, 8, 2] that processes 400 g of starting material in order to reduce extraction biases [9]. Triplicate PCRs were prepared in 20 µl reactions containing of 2 µl template, 1.5 mM $MgCl_2$, 1 x AmpliTaq Gold buffer (Thermo Fisher Scientific, Waltham, US-MA), 0.25 mM dNTPs, 0.5 µM forward and reverse primer and 1.25 U AmpliTaq Gold (Thermo Fisher Scientific, Waltham, US-MA). Thermal cycling was performed with with initial denaturation at 94 °C (3 min), followed by 35 cycles (94 °C for 45 s, 57 °C for 1 min, and 72 °C for 1:30 min), followed by a final elongation of 10 min (72 °C). Long extension times were used to counteract chimera formation [10, 11]. Combined and un-quantified replicate amplicons were purified using Agencourt AMPure XP (Beckman Coulter, Brea, US-CA), quantified using Qubit QuantiFluor dsDNA kits (Promega, Fitchburg, US-WI). Amplicons above 0.25 ng/µl were then pooled by weight (Fig. 3). Concentrations in library pools were measured using a 2100 Bioanalyser (Agilent Technologies, Santa Clara, US-CA) and diluted to 9 pM for sequencing. Libraries were sequenced in both directions on an Illumina MiSeq with 300 cycles (Illumina, San Diego, US-CA) using a reagents kit v2 to retrieve 150 bp paired-end reads. DNA extraction and PCR controls were included into amplification and sequencing for both genes if the cleaned amplicons were sufficiently concentrated. Among 192 combined PCR reactions for 18S, six of 13 PCR controls and one of three blended extraction controls were sequenced (Fig. 3).

1.4 Deconvolution and chimera screening

Deconvolution and chimera screening were performed with QIIME 1.9. [12]. Deconvolution was accomplished using `split_libraries.py` with parameters `-q 19` (maximum unacceptable Phred score). The effect of chimeras in metataxonomic data is detrimental [13]. Chimera screening of unclustered 18S data was performed in a *de-novo* approach using USEARCH 6.1 [14] with `identify_chimeric_seqs.py`. Additionally, to reduce the effect of chimeras and other sequence artefacts, and to allow more reliable abundance correction, phylotypes with abundances less then 100 sequences and samples with less then 1000 sequences were removed at a later stage (see below), similar to approaches outlined elsewhere [15, 16].

1.5 Reference data and phylotype definition

Phylotypes were defined as described in [2] using `pick_otus.py` with additional reverse strand matching `-z`. A clustering threshold of 97% was chosen. SILVA reference data [17] v119 was used for taxonomic assignments with `assign_taxonomy.py`, a taxonomy assignment threshold of 90% was chosen.

1.6 Filtering and abundance correction

Phylotypes from sequenced positive and negative controls, phylotypes with abundances less then 100 sequences and samples with less then 1000 sequences were removed from the sequence data using `filter_otus_from_otu_table.py` and `filter_samples_from_otu_table.py` with corresponding filter criteria. Sequence data was then abundance-corrected using cumulative sum scaling (CSS) [18] as implemented in `normalize_table.py`.

2 Data and analysis scripts

The documented code to conduct analyses is maintained at `https://github.com/macrobiotus/antarctic_invertebrates.git`, the release used here is available via `http://doi.org/10.5281/zenodo.190926`. R objects and sequence information used here are available via `http://doi.org/10.5281/zenodo.162484`.

References

[1] Clarke, L. J., Soubrier, J., Weyrich, L. S. & Cooper, A. Environmental metabarcodes for insects: in silico PCR reveals potential for taxonomic bias. *Molecular Ecology Resources* **14**, 1160–1170 (2014). URL `http://doi.wiley.com/10.1111/1755-0998.12265`.

[2] Czechowski, P., Clarke, L. J., Breen, J., Cooper, A. & Stevens, M. I. Antarctic eukaryotic soil diversity of the Prince Charles Mountains revealed by high-throughput sequencing. *Soil Biology and Biochemistry* **95**, 112–121 (2016). URL `http://dx.doi.org/10.1016/j.soilbio.2015.12.013`.

[3] Gilbert, J. A. *et al.* Meeting Report: The Terabase Metagenomics Workshop and the Vision of an Earth Microbiome Project. *Standards in Genomic Sciences* **3**, 243–248 (2010). URL `http://www.standardsingenomics.org/index.php/sigen/article/view/sigs.1433550`.

[4] Parfrey, L. W. *et al.* Communities of microbial eukaryotes in the mammalian gut within the context of environmental eukaryotic diversity. *Frontiers in Microbiology* **5**, 1–13 (2014). URL `http://journal.frontiersin.org/article/10.3389/fmicb.2014.00298/abstract`.

[5] Golay, M. J. E. Notes on digital coding. *Proceedings of the Institute of Radio Engineers* **37**, 657 (1949).

[6] Pankhurst, C. E., Ophel-Keller, K., Doube, B. M. & Gupta, V. V. S. R. Biodiversity of soil microbial communities in agricultural systems. *Biodiversity and Conservation* **5**, 197–209 (1996). URL `http://link.springer.com/10.1007/BF00055830`.

[7] Ophel-Keller, K., McKay, A., Hartley, D., Herdina, . & Curran, J. Development of a routine DNA-based testing service for soilborne diseases in Australia. *Australasian Plant Pathology* **37**, 243 (2008). URL `http://link.springer.com/10.1071/AP08029`.

[8] Huang, C. Y. *et al.* A DNA-based method for studying root responses to drought in field-grown wheat genotypes. *Scientific Reports* **3**, 1–7 (2013). URL `http://www.nature.com/doifinder/10.1038/srep03194`.

[9] TABERLET, P. *et al.* Soil sampling and isolation of extracellular DNA from large amount of starting material suitable for metabarcoding studies. *Molecular Ecology* **21**, 1816–1820 (2012). URL `http://doi.wiley.com/10.1111/j.1365-294X.2011.05317.x`.

[10] Lenz, T. L. & Becker, S. Simple approach to reduce PCR artefact formation leads to reliable genotyping of MHC and other highly polymorphic loci - Implications for evolutionary analysis. *Gene* **427**, 117–123 (2008). URL `http://linkinghub.elsevier.com/retrieve/pii/S037811190800471X`.

[11] Yu, D. W. *et al.* Biodiversity soup: metabarcoding of arthropods for rapid biodiversity assessment and biomonitoring. *Methods in Ecology and Evolution* **3**, 613–623 (2012). URL `http://doi.wiley.com/10.1111/j.2041-210X.2012.00198.x`.

[12] Caporaso, J. G. *et al.* QIIME allows analysis of high-throughput community sequencing data. *Nature Methods* **7**, 335–336 (2010). URL `http://www.nature.com/doifinder/10.1038/nmeth.f.303`.

[13] Edgar, R. C. UPARSE: Highly accurate OTU sequences from microbial amplicon reads. *Nature Methods* **10**, 996–998 (2013).

[14] Edgar, R. C. Search and clustering orders of magnitude faster than BLAST. *Bioinformatics* **26**, 2460–2461 (2010). URL `http://bioinformatics.oxfordjournals.org/cgi/doi/10.1093/bioinformatics/btq461`.

[15] Carew, M. E., Pettigrove, V. J., Metzeling, L. & Hoffmann, A. a. Environmental monitoring using next generation sequencing: rapid identification of macroinvertebrate bioindicator species. *Frontiers in Zoology* **10**, 45 (2013). URL http://frontiersinzoology.biomedcentral.com/articles/10.1186/1742-9994-10-45.

[16] Blaalid, R. *et al.* ITS1 versus ITS2 as DNA metabarcodes for fungi. *Molecular Ecology Resources* **13**, 218–224 (2013).

[17] Pruesse, E. *et al.* SILVA: a comprehensive online resource for quality checked and aligned ribosomal RNA sequence data compatible with ARB. *Nucleic Acids Research* **35**, 7188–7196 (2007). URL http://nar.oxfordjournals.org/lookup/doi/10.1093/nar/gkm864.

[18] Paulson, J. N., Stine, O. C., Bravo, H. C. & Pop, M. Differential abundance analysis for microbial marker-gene surveys. *Nature Methods* **10**, 1200–1202 (2013). URL http://www.nature.com/doifinder/10.1038/nmeth.2658. NIHMS150003.

[19] Caporaso, J. G. *et al.* Ultra-high-throughput microbial community analysis on the Illumina HiSeq and MiSeq platforms. *The ISME Journal* **6**, 1621–1624 (2012).

[20] Rayment, G. E. & Lyons, D. J. *Soil Chemical Methods - Australasia* (CSIRO publishing, Collingwood, 2011).

3 Supplemental figures

	1	2	3	4	5	6	7	8	9	10	11	12
A	AC24981<br>Reinbolt	AC23007<br>Hills	AC23008	AC23009	AC23010	AC23011	AC08310<br>Mount	AC26651<br>Menzies	AC08312	AC08313	AC08314	PCR (-)
B	AC26652	AC08315	AC08316	AC08317	AC26623	AC08319	AC26619	AC08320	AC08323	AC08324	AC26616	PCR (-)
C	AC08325	AC24977	AC08326	AC08327	AC26633	AC08328	AC23016		AC26625	AC08332	AC26645	PCR (-)
D	AC08340<br>Mawson	AC29096<br>Escarpment	AC29038	AC08341	AC29039	AC29093	AC08343	AC26644	AC29097	AC29049	AC29043	PCR (-)
E	AC29045	AC08347	AC29046	AC29048	AC23022	AC29099	AC23023	AC08351	AC29102	AC29050	AC24980	PCR (-)
F	AC29051	AC08353	AC29103	AC26626	AC23025	AC23024	AC08355	AC29104	AC08356	AC29052	AC29105	PCR (-)
G	AC29106	AC08358	AC29053	AC29107	AC29130	AC29054	AC08361	AC29108	AC08362	AC29132	AC08363	PCR (-)
H	AC29055	AC08364	AC29056	AC08365	AC29110	AC08366	AC24982	AC23026	AC08367	AC08401<br>soil cntrl 1	AC29126<br>soil cntrl 2	PCR (-)

	1	2	3	4	5	6	7	8	9	10	11	12
A	AC29111	AC29112	AC08368	AC29113	AC29131	AC23027	AC29128	AC29057	AC23030	AC24957	AC29114	PCR (NTC)<br>#3920 #3972
B	AC23028	AC29115	AC08441	AC29116	AC23035	AC23031	AC08464	AC08371<br>Amery	AC08475<br>Oasis	AC24959	AC08372	PCR (NTC)<br>#3973 #4062
C	AC08373	AC24958	AC23039	AC26621	AC08375	AC26620	AC08376	AC23038	AC08377	AC26629	AC26649	PCR (NTC)<br>#4200 #4201
D	AC08378	AC24961	AC08379	AC26648	AC24962	AC08380	AC24978	AC08381	AC26647	AC08382	AC26617	PCR (-)
E	AC08383	AC24963	AC26636	AC26624	AC08494	AC08385	AC24965	AC08384	AC08386	AC26640	AC26618	PCR (-)
F	AC08388	AC08389	AC24970	AC08390	AC24968	AC24969	AC29061	AC29062	AC08394	AC29063	AC08395	PCR (-)
G	AC29064	AC08398	AC29069	AC29070	AC29072	AC29074	AC29075	AC24972	AC24976 in via	AC29077	AC29118	PCR (-)
H	AC29123	AC29041	AC08413 R<br>3920-2 E11	AC08427 T<br>3920-2 A3	AC08416 N<br>3920-2 E6	AC08407 M<br>3920-D3	H3 - H6<br>mixed 28.12.1	insect blend<br>ins cntrl 1 (+)	damselfly<br>ins cntrl 2 (+)	AC08401<br>soil cntrl 1(+)	AC29126<br>soil cntrl 2 (+)	PCR (-)

Figure 1: DNA extracts allocation on micro-titre plates. Sample origins were indicated with shades of yellow, given were extract identifiers ("AC"). Sample names used in this study corresponded to plate positions (ranging from 1.1.A to 2.12.H.; for plate 1, well 1A, to plate 2, well 12H, respectively). Row 12 of each plate contained pools of extract controls or PCR controls (H_2O prior to amplification). Wells 10H and 11H on plate 1 and wells 3H –11H of plate 2 contained control DNA extracts, of which wells 8H, 10H and 11H were analysed in another project. Additionally, phylotypes obtained from wells 10H and 11H were used for the retention of Antarctic invertebrates as detailed in subsection 1.6.

18S primers

rev

stock solution	
[M] stock	
add [n] µl TE	
stock [C]	10

working dilution	
use [n] µl stock	5
use [n] µl water	5
[M] working dilution	5

work plate
5 ul H2O + 5 ul rev each well = 10 ul rev
add 10 ul fwd -- 20 ul each well

18S_1_wd	1	2	3	4	5	6	7	8	9	10	11	12
A	AC24981	AC23007	AC23008	AC23009	AC23010	AC23011	AC08310	AC26651	AC08312	AC08313	AC08314	PCR (-)
	EukBr_Hiseq_04	412	413	414	415	416	417	418	419	420	421	422
B	AC26652	AC08315	AC08316	AC08317	AC26623	AC08319	AC26619	AC08320	AC08323	AC08324	AC26616	PCR (-)
	423	424	425	426	427	428	429	430	431	432	433	434
C	AC08325	AC26634	AC08326	AC08327	AC26633	AC08328	AC23016	AC08331	AC26625	AC08332	AC26645	PCR (-)
	435	436	437	438	439	440	441	442	443	444	445	446
D	AC08340	AC29096	AC29038	AC08341	AC29039	AC29093	AC08343	AC26644	AC29097	AC29049	AC29043	PCR (-)
	447	448	449	450	451	452	453	454	455	456	457	458
E	AC29045	AC08347	AC29046	AC29048	AC23022	AC29099	AC23023	AC08351	AC29102	AC29050	AC24980	PCR (-)
	459	460	461	462	463	464	465	466	467	468	469	470
F	AC29051	AC08353	AC29103	AC26626	AC23025	AC23024	AC08355	AC29104	AC08356	AC29052	AC29105	PCR (-)
	471	472	473	474	475	476	477	478	479	480	481	482
G	AC29106	AC08358	AC29053	AC29107	AC29130	AC29054	AC08361	AC29108	AC08362	AC29132	AC08363	PCR (-)
	483	484	485	486	487	488	489	490	491	492	493	494
H	AC29055	AC08364	AC29056	AC08365	AC29110	AC08366	AC24982	AC23026	AC08367	AC08401	AC29126	PCR (-)
	495	496	497	498	499	500	501	502	503	504	505	506

used		
	yellow capped vial	10
	purple plates	4
	8 stip tubes	6

rev

stock plate	
[nM] stock	25
add [n] µl TE	250
stock [µM]	100
volume [µl]	250

work plate	
use [n] µl stock	5
use [n] µl water	95
working dilution C [µM]	5
volume [µl]	100

dilution_plate
add 10 ul rev
add 10 ul fwd
20 ul each well

18S_2_stock /v	1	2	3	4	5	6	7	8	9	10	11	12	
A	AC29111	AC29112	AC08368	AC29113	AC29131	AC23027	AC29128	AC29057	AC23030	AC24957	AC29114	PCR (-)	
	EukBr_Hiseq_0C	18	19	20	21	22	23	24	25	26	27	28	
B	AC23028	AC29115	AC23029	AC29116	AC23035	AC23031	AC23032	AC08371	AC23040	AC24959	AC08372	PCR (-)	
	29	30	31	32	33	34	35	36	37	38	39	40	
C	AC08373	AC24958	AC23039	AC26621	AC08375	AC26620	AC08376	AC23038	AC08377	AC26629	AC26649	PCR (-)	
	41	42	43	44	45	46	47	48	49	50	51	52	
D	AC08378	AC24961	AC08379	AC26648	AC24962	AC08380	AC24978	AC08381	AC26647	AC08382	AC26617	PCR (-)	
	53	54	55	56	57	58	59	60	61	62	63	64	
E	AC08383	AC24963	AC26636	AC26624	AC23041	AC08385	AC24965	AC08384	AC08386	AC26640	AC26618	PCR (-)	
	65	66	67	68	69	70	71	72	73	74	75	76	was 78
F	AC08388	AC08389	AC24970	AC08390	AC24968	AC24969	AC29061	AC29062	AC08394	AC29063	AC08395	PCR (-)	
	77	78	79	80	81	82	83	84	85	86	87	88	
G	AC29064	AC08398	AC29069	AC29070	AC29072	AC29074	AC29075	AC24972	AC29076	AC29077	AC29118	PCR (-)	
	89	90	91	92	93	94	95	96	97	98	99	100	
H	AC29123	AC29041	AC08413 R	AC08427 T	AC08416 N	AC08407 M H3 - H6 mixed		insect blend (+)	damselfly (+)	soil 1 (+)	soil2 (+)	PCR (-)	
	101	102	103	104	105	106	107	108	109	110	111	112	

fwd 18S Euk

stock solution	
[nM] stock	32.9
add [n] µl TE	329
stock C [µM]	100

working dilution	
use [n] µl stock	100
use [n] µl water	1900
working dilution [µM]	5
eeded volume 96 x 2 x 10ul	2000
strip tube: 2000 / 8 =	250
add fwd to each well	10

Figure 2: Fusion primers employed for sequencing of 18S amplicons on the Illumina platform. Detailed primer design was described in detail elsewhere [2], numbers over wells refer to barcode identifiers in [19].

not sequenced
sequenced

18 plate 1 **(ng / µl)**

	1	2	3	4	5	6	7	8	9	10	11	12	
**A**	11.68	19.28	14.83	16.70	10.53	21.85	2.50	6.28	1.65	3.53	1.55	0.43	PCR (-)
**B**	2.63	1.55	19.30	4.90	11.90	2.10	4.08	7.33	2.10	1.18	5.28	0.75	PCR (-)
**C**	0.95	9.65	0.68	0.85	1.93	1.00	20.10	0.38	0.05	1.00	11.88	2.63	PCR (-)
**D**	11.43	6.20	13.13	7.75	5.60	12.28	6.73	6.03	16.10	11.43	8.83	1.73	PCR (-)
**E**	2.93	6.95	11.30	9.18	6.55	13.85	1.08	1.65	9.45	5.75	12.88	0.48	PCR (-)
**F**	10.03	5.00	2.53	5.23	14.95	8.30	8.60	11.53	2.20	12.53	0.63	0.45	PCR (-)
**G**	5.98	13.40	0.45	0.73	0.05	0.68	10.00	3.00	11.55	11.95	16.63	1.80	PCR (-)
**H**	5.88	16.43	0.55	0.68	8.18	10.35	19.05	16.28	2.38	15.63	19.25	0.70	PCR (-)
										AU (+)	AU (+)		

18 plate 2 **(ng / µl)**

	1	2	3	4	5	6	7	8	9	10	11	12	
**A**	3.90	3.15	13.98	1.73	1.30	7.80	8.13	6.00	2.80	5.33	0.35	1.03	XTR (-) pool
**B**	2.45	3.90	7.10	4.83	7.25	5.48	7.45	6.85	9.03	3.18	3.23	0.51	XTR (-) pool
**C**	2.03	2.40	0.30	4.85	3.25	7.30	7.10	5.35	3.93	0.13	5.90	0.49	XTR (-) pool
**D**	1.35	1.15	8.33	5.68	5.13	3.05	5.00	5.03	3.05	0.78	3.38	0.13	PCR (-)
**E**	1.58	4.73	1.98	3.55	4.38	1.28	2.95	4.10	2.43	0.13	1.80	0.33	PCR (-)
**F**	0.80	0.68	2.78	6.28	4.20	1.83	2.43	2.68	4.68	3.05	3.20	0.42	PCR (-)
**G**	0.85	2.10	3.95	2.38	4.48	0.18	0.28	2.88	4.38	4.48	0.25	0.78	PCR (-)
**H**	0.88	1.50	5.40	5.65	6.93	0.65	7.23	6.65	11.10	9.73	10.00	0.13	PCR (-)
			R (+)	T (+)	N (+)	M (+)	RTNM (+)	INS1 (+)	INS2 (+)	AU1 (+)			

Figure 3: Concentrations of purified DNA pools after combining of three PCR replicates. Equimolar pools were used for sequencing on the Illumina platform. Plate positions marked in green indicate sufficient concentration for pooling, positions marked in red were insufficiently concentrated for pooling. PCR control reactions are marked "PCR (-)". Pools of extraction controls are marked "XTR (-) pool". Several positive controls were included: Two extracts of two different Australian soils are marked "AU1 (+)" and "AU2(+)", respectively. An artificial pool of Austrlian insects, and a pure extract of Damselfly (see [1]) are marked "INS1(+)" and "INS2(+)", respectivly. Antarctic bulk soil extracts, containing rotifers, tardigrades, nematodes and mites in high abundance (determined by morphological examination) are marked with "R(+)", "T(+)", "N(+)" and "M(+)", respectivly. A pool of these fromer four extracts is marked with "RTMN(+)".

4 Supplemental tables

Table 1: Extraction methods employed for soil geochemical analysis, provided by CSBP Soil and Plant Analysis Laboratory (Bibra Lake, AU-QLD) and referenced in [20].

Analytes	Method, Unit, Detection Limit
P and K	Method 9B1; Unit: mg/kg; Limits: 2
Soil pH in $CaCl_2$	Method 4B1; Unit: pH; Limits: 2 decimal points
Soil pH in H_2O	Method 4A1; Unit: pH; Limits: 2 decimal points
Organic C	Method 6A1; Unit: %; Limits: 0.05
NH_4^+ and NO_3^- N	Method 7C2; Unit: mg/kg; Limits: 1
KCl S	Method 10D1; Unit: mg/kg; Limits: 0.5
electric conductivity	Method 3A1; Unit: dS/m; Limits: 0.01
P	Method 9C2; Unit: mg/kg; Limits: 0.1

Main analysis

Paul Czechowski

December 5th, 2016

Contents

Preface **2**

Prerequisites to run this analysis **2**

Environment preparation **2**
Package loading and cleaning of work-space . . . 2
Setting locations for data import and export . . . 2
Import locations . . . 3
Export locations . . . 3
Loading functions . . . 3
Data import . . . 4
Defining predictor categories . . . 4

Describe imported `phyloseq` object **4**
Invertebrate abundances per sample . . . 4
Invertebrate abundances per location . . . 6
Map in addition to QGIS map . . . 8

Retrieve and write information from imported `phyloseq` object **9**
Mean elevation per sample . . . 9
Write sample coordinates to be used with GIS software . . . 9

Saving intermediate workspace **10**

Exploratory data analysis **10**
Isolating data for this analysis . . . 10
Checking soil mineral and soil geochemical data . . . 10
Showing the initial state of the mineral and chemical data . . . 10
Removing outliers in soil geochemical observations . . . 13
Transformations . . . 13
Plotting correlations . . . 15
Removing the correlated variables . . . 17
Plotting out transformed mineral and chemical data . . . 17
Principal component analysis of observations . . . 17
Getting the principal components . . . 17
Testing the principal components . . . 20
Bi-plot . . . 20
Checking species information . . . 20
Showing the initial state of the species information . . . 20

Non-Metric Multidimensional Scaling **22**
Matching up phylotype and factor information. . . . 22
Getting a `metaMDS` object from the species data . . . 22
Fitting environmental vectors . . . 25
`adonis` analysis . . . 26
Canonical correspondence analysis . . . 28

Calculate the CCA . . . 28
Testing CCA results . . . 28
Regression analyses . . . 30
Across all samples . . . 30
Region specific . . . 35

Write data to disk **49**

Session info **49**

References **50**

Preface

This text was re-rendered in the proofing stage, it is slightly newer then the versions available in the released online repositories. This code is tested using a raw R terminal. Path names are defined relative to the project directory. This code commentary is included in the R code itself and can be rendered at any stage using `rmarkdown::render ("40_main_analysis.r")`. Please check the session info at the end of the document for further notes on the coding environment.

Prerequisites to run this analysis

- This script is in the parent directory of the repository.
- Scripts `10_import_predictors.r`, `20_format_predictors.r`, and `30_format_phyloseq.r`were run.
- Script `00_functions.r` is available .
- Output of these scripts is available throughout the repository directory tree.

Environment preparation

Package loading and cleaning of work-space

```
library ("phyloseq")   # required for working with phyloseq objects
library ("ggbiplot")   # package for simple generation of PCA biplots
library ("ggplot2")    # package used for barplot (for themes())
library ("corrplot")   # plotting of correlations ...
library ("ggcorrplot") # ... which can be saved to disk
library ("gridExtra")  # re-arranging graphical objects
library ("GGally")     # scatter plot matrices (geochemical and combined data)
library ("vegan")      # CCA and MDS
library ("dplyr")      # for easier reading of complex expressions
rm(list=ls())          # clear R environment
                       # working directory needs to be set manually for
                       #   cross-platform compatibility
```

Setting locations for data import and export

This script uses the objects generated by `30_format_phyloseq.r` that are located in the `Zenodo` directory tree. It will also write to that location. The number in front of the file name denotes the source script.

Import locations

Path to filtered, CSS abundance-corrected data with curated taxonomy and field measurements.

```
path_phsq_ob <- file.path ("Zenodo/R_Objects/35_phsq_ob.Rdata",
                           fsep = .Platform$file.sep)
```

Export locations

Workspace image for future reference, after plotiing and after analyses.

```
path_workspace_a    <- file.path ("Zenodo/R_Objects/40_010_workspace.Rdata",
                                  fsep = .Platform$file.sep)

path_workspace_b    <- file.path ("Zenodo/R_Objects/40_020_workspace.Rdata",
                                  fsep = .Platform$file.sep)
```

Graphics for main manuscript and supplemental information.

```
path_abnds   <- file.path ('Zenodo/R_Output/40-010_abundances.pdf',
                          fsep = .Platform$file.sep)
path_coord   <- file.path ('Zenodo/R_Output/40-020_coordinates.csv',
                          fsep = .Platform$file.sep)
path_brplts  <- file.path ('Zenodo/R_Output/40-030_barplots.pdf',
                          fsep = .Platform$file.sep)
path_map_pcm <- file.path ('Zenodo/R_Output/40-040_map_pcm.pdf',
                          fsep = .Platform$file.sep)
path_all_cor <- file.path ('Zenodo/R_Output/40-050_cor_all_vars.pdf',
                          fsep = .Platform$file.sep)
path_min_cor <- file.path ('Zenodo/R_Output/40-060_cor_min_vars.pdf',
                          fsep = .Platform$file.sep)
path_sca_tra <- file.path ('Zenodo/R_Output/40-070_sca_tra_all_vars.pdf',
                          fsep = .Platform$file.sep)
path_vio_tra <- file.path ('Zenodo/R_Output/40-080_vio_tra_all_vars.pdf',
                          fsep = .Platform$file.sep)
path_pca_var <- file.path ('Zenodo/R_Output/40-090_pca_var_all_vars.pdf',
                          fsep = .Platform$file.sep)
path_pca_bip <- file.path ('Zenodo/R_Output/40-110_pca_bip_all_vars.pdf',
                          fsep = .Platform$file.sep)
path_mds_hip <- file.path ('Zenodo/R_Output/40-120_mds_mds_all_vars.pdf',
                          fsep = .Platform$file.sep)
path_hmp_all <- file.path ('Zenodo/R_Output/40-130_hmp_____all_vars.pdf',
                          fsep = .Platform$file.sep)
path_regr    <- file.path ('Zenodo/R_Output/40-140_regr_age_chem.pdf',
                          fsep = .Platform$file.sep)
```

Loading functions

This script uses many functions, and it impractical to have them all in here. They are loaded from `00_functions.R`.

```
source (file.path ("00_functions.r", fsep = .Platform$file.sep))
```

Data import

Phylotype data is imported using basic R functionality. Imported are `phyloseq` objects (McMurdie and Holmes 2013).

```
load (path_phsq_ob) # object name is "phsq_ob"
```

Defining predictor categories

These character vectors can be passed to `get_predictors()` to create data frames with customised variable content.

```
geochems <- c ("AMMN", "NITR", "POTA", "SLPH", "COND", "PHCC",  "PHOS", "CARB",
               "PHHO")
                        # "AMMN" and "NITR" with 57% and 47% undefined data
                        #   may be removed, these may hold important
                        #   information. "PHOS" "CARB" may be removed because not
                        #   relevant for MM?
minerals <- c ("QUTZ", "FDSP", "TTAN", "PRAG", "MICA", "DOLO", "KAOC")
                        # "CALC" with 33 NA's and "CHLR" with 52 NA's
                        #   are removed, otherwise the MM mineral composition
                        #   can't be analysed
location <- c ("AREA")
position <- c ("LONG", "LATI") # for spatial distance matrices
raw_ages <- c ("LAGE", "HAGE") # low and high age estimate
```

Describe imported `phyloseq` object

Invertebrate abundances per sample

The following plots are agglomerated on a specified level by calling `agglomerate()`. The first plot shows proportional abundance of invertebrates in rarefied data. In the second plot, all abundances are converted to "1", (via `make_binary ()`)and the rank composition is more visible. Create the plots.:

```
pl1 <- barplot_samples (agglomerate (phsq_ob, "Class"), "Class")
pl2 <- barplot_samples (make_binary (agglomerate (phsq_ob, "Class")), "Class")
```

Showing the plots.:

```
grid.arrange(pl1, pl2, nrow = 2)
```

Save the plots.:

```
ggsave (file = path_abnds, plot = arrangeGrob (pl1, pl2, nrow = 2),
        dpi = 200, width = 7, height = 7, units = "in")
```

Garbage collection.

```
rm(pl1, pl2)
```

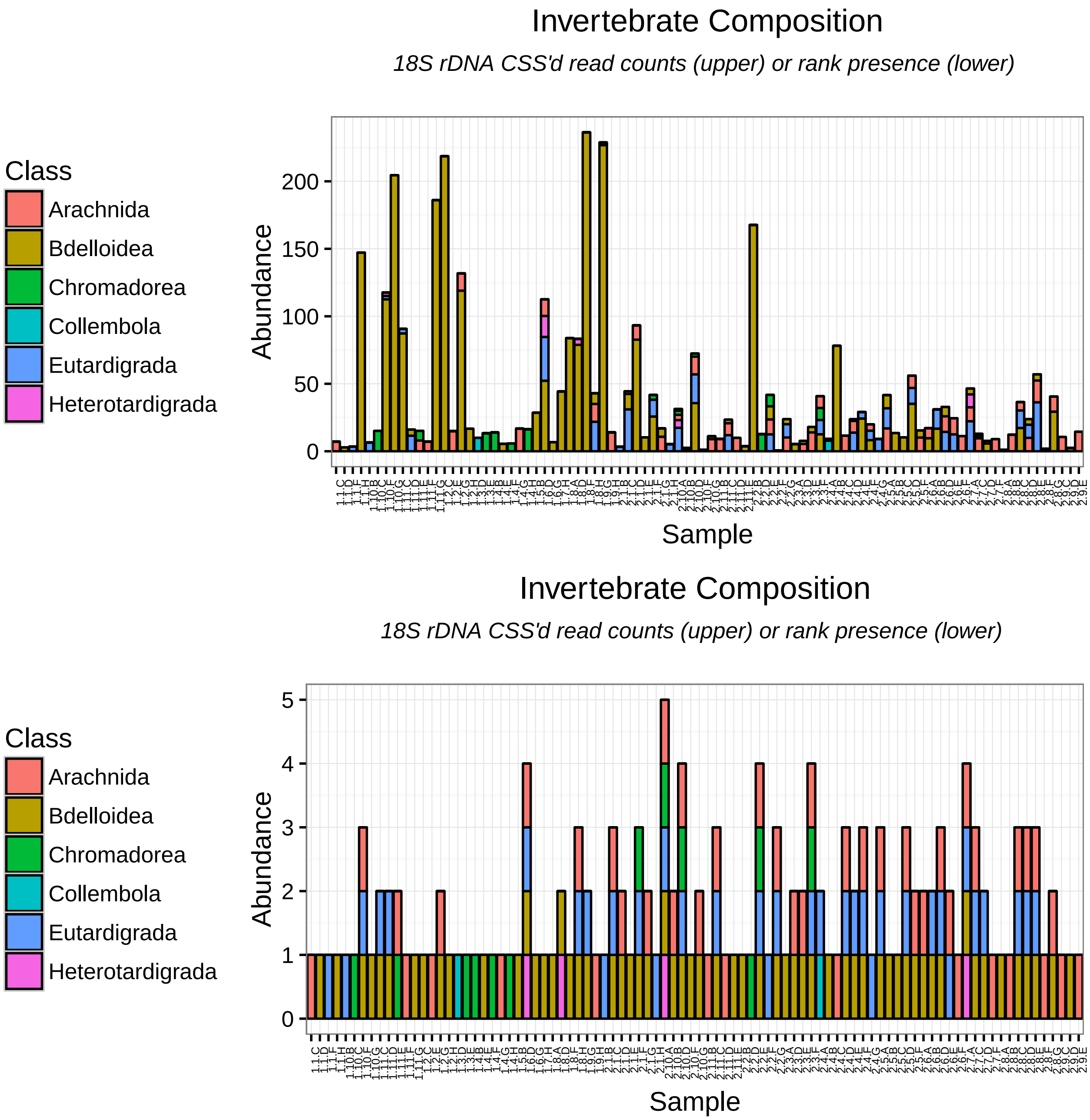

Figure 1: Invertebrate abundances at selected taxonomic level. (approx. code line 149)

Invertebrate abundances per location

Bar-plot taxa by locations. Firstly, building a list which can be used to give and take from plotting function. "[[ ]]" will get the atomic vector rather then a sub-data frame, to give a character vector of length three (3 locations).

```
areas <- unique (as.character (as.data.frame (sample_data (phsq_ob))[["AREA"]]))
```

An object will be agglomerated on class level to match the previous plots.

```
phsq_agg <- agglomerate (phsq_ob, "Class")
```

`subset_samples()` and `lapply()` are still not friends, but subsetting per location is still necessary.

```
phsq_obs <- list (subset_samples (phsq_ob,
                                  sample_data (phsq_ob)[, "AREA"] == areas[[1]]),
                  subset_samples (phsq_ob,
                                  sample_data (phsq_ob)[, "AREA"] == areas[[2]]),
                  subset_samples (phsq_ob,
                                  sample_data (phsq_ob)[, "AREA"] == areas[[3]]))
```

I will also filter for empty sample columns, as no empty are columns wanted in plots.

```
phsq_obs <- lapply (phsq_obs, remove_empty)
```

Looks like `plot_bar()` doesn't like `lapply()` either, as it looks, so I need to create a plot for each location individually.

```
plots <- list (plot_bar (phsq_obs[[1]], fill = "Class", facet_grid = "Phylum~AREA") +
                 theme_bw() + theme(axis.text.x = element_text(angle = 90, hjust = 1,
                 size = 8), strip.text.x = element_text(size = 12),
                 strip.text.y = element_text(size = 12, angle = 0),
                 axis.text.y = element_text (size = 8)) +
                 geom_bar ( aes(color = Class, fill = Class), stat="identity",
                 position="stack"),
               plot_bar (phsq_obs[[3]], fill = "Class", facet_grid = "Phylum~AREA") +
                 theme_bw() + theme(axis.text.x = element_text(angle = 90, hjust = 1,
                 size = 8), strip.text.x = element_text(size = 12),
                 strip.text.y = element_text(size = 12, angle = 0),
                 axis.text.y = element_text (size = 8)) +
                 geom_bar ( aes(color = Class, fill = Class), stat="identity",
                 position="stack"),
               plot_bar (phsq_obs[[2]], fill = "Class", facet_grid = "Phylum~AREA") +
                 theme_bw() + theme(axis.text.x = element_text(angle = 90, hjust = 1,
                 size = 8), strip.text.x = element_text(size = 12),
                 strip.text.y = element_text(size = 12, angle = 0),
                 axis.text.y = element_text (size = 8)) +
                 geom_bar ( aes(color = Class, fill = Class), stat="identity",
                 position="stack"))
```

Plots can now be shown, and saved to the output directory. Objects are then discarded.

```
grid.arrange(plots[[1]], plots[[2]], plots[[3]], nrow = 3)
```

Saving plots.:

```
ggsave (file = path_brplts, plot = arrangeGrob (plots[[1]], plots[[2]],
                                                plots[[3]], nrow = 3),
        dpi = 200, width = 7, height = 10, units = "in")
```

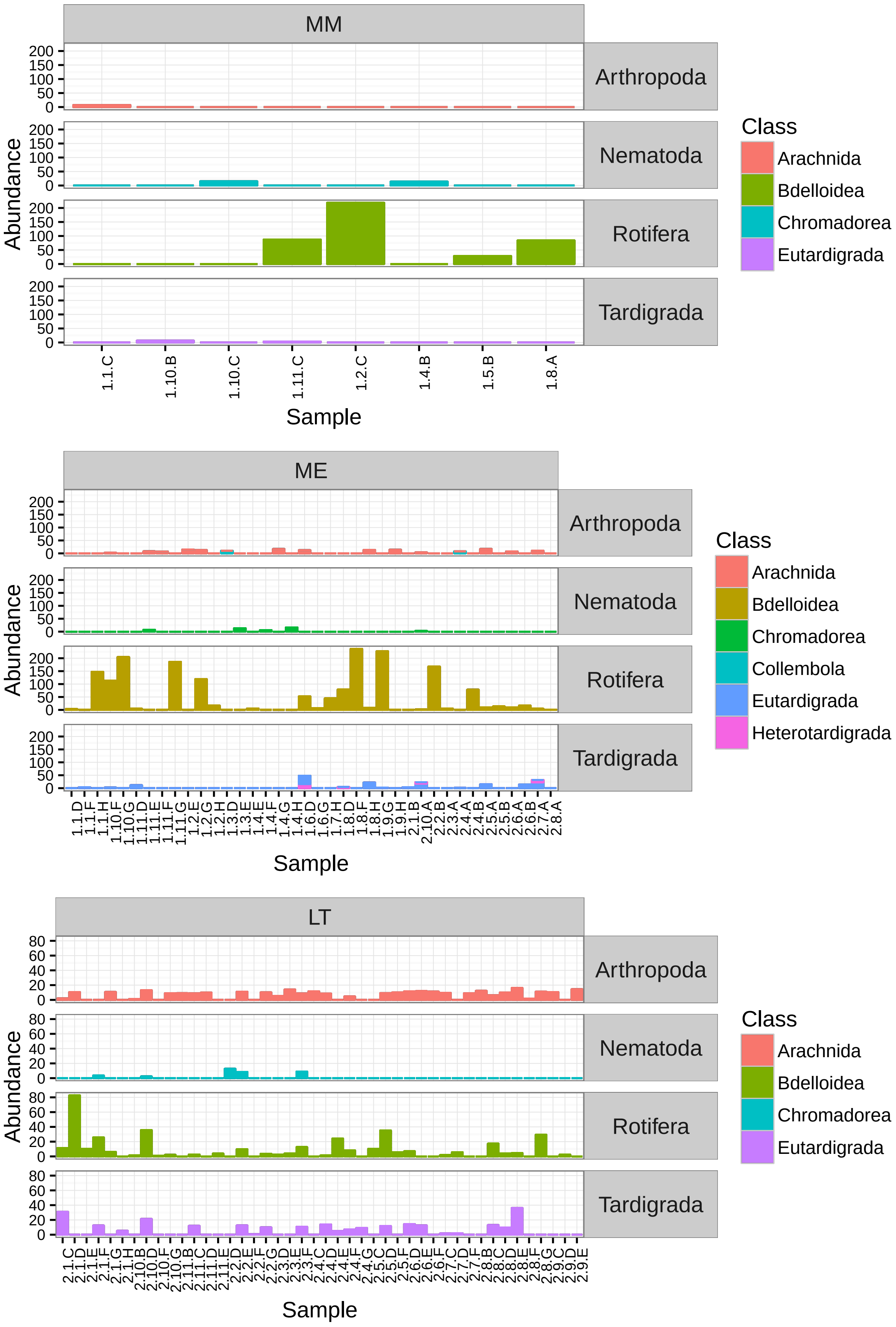

Figure 2: Invertebrate class and phylum composition per sampling location, with CSS'd abundances. Note: This is not the same figure as in the main text. (Approx. code line 209)

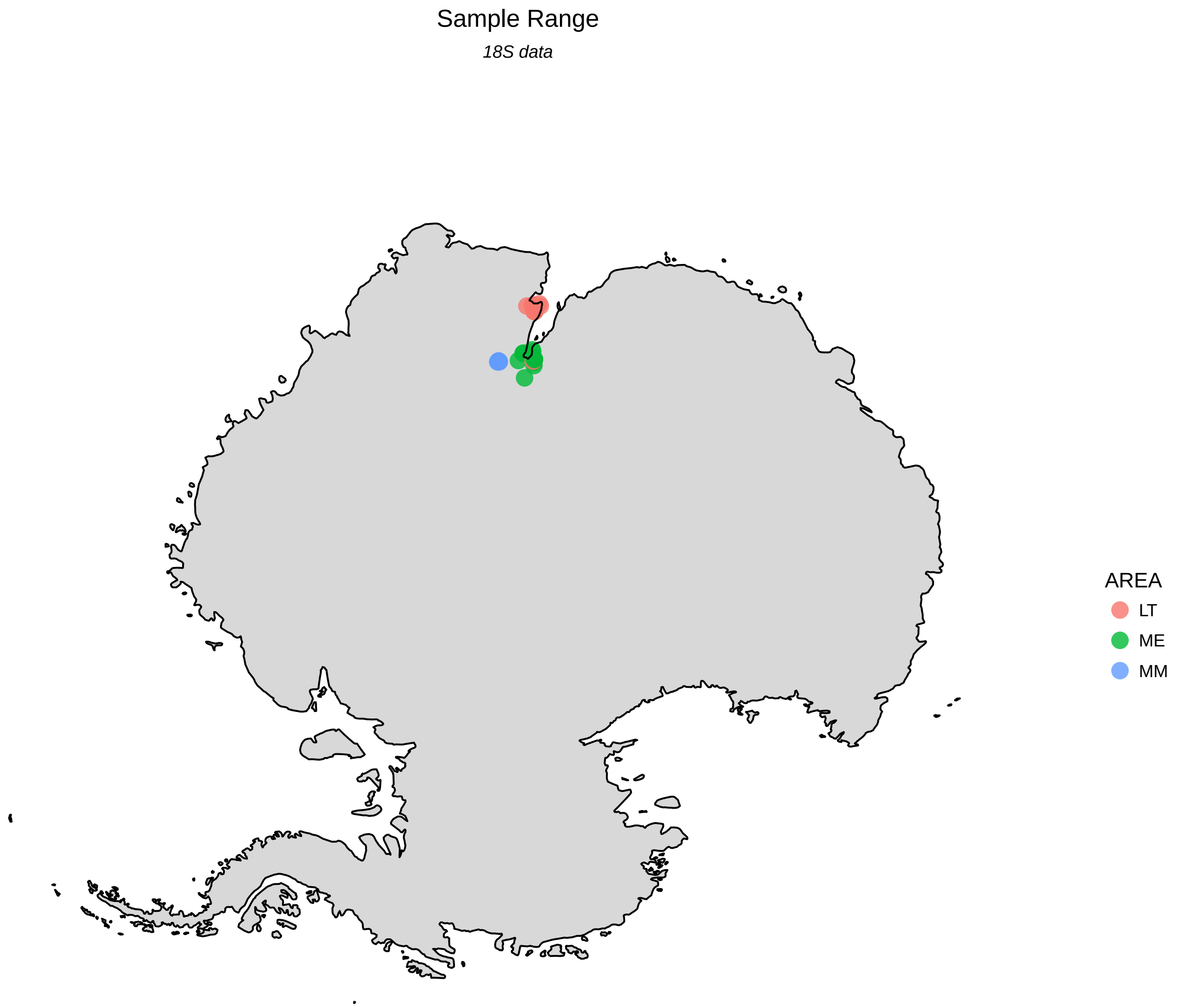

Figure 3: Sample range of responses and predictors (approx. code line 228)

Garbage collection.:

```
rm (plots, phsq_obs, phsq_agg)
```

Map in addition to QGIS map

Here using an old function from repository `pcm_modelling`, which was minimally adjusted for the current repository. Creating the map, plotting is here, and saving it to file. Garbage collection afterwards.

```
phsq_map <- map_samples(phsq_ob, col_long = "LONG", col_lat = "LATI",
  subtitle = "18S data")
```

```
plot(phsq_map)
```

```r
ggsave (path_map_pcm, plot = phsq_map, dpi = 200, width = 5.5, height = 4.5,
  units = "in")

rm (phsq_map)
```

Retrieve and write information from imported `phyloseq` object

Expand this section if further information is necessary for writing.

Mean elevation per sample

Get elevation range for locations:

```r
temp <- data.frame (sample_data (phsq_ob)[ , c("ELEV", "AREA")])
```

Output mean elevations per location:

```r
summary (temp [ which (temp$AREA == "MM"),  "ELEV" ]) # MM
```

```
##    Min. 1st Qu.  Median    Mean 3rd Qu.    Max.    NA's
##    1614    1693    1828    1796    1898    1950       1
```

```r
summary (temp [ which (temp$AREA == "ME"),  "ELEV" ]) # ME
```

```
##    Min. 1st Qu.  Median    Mean 3rd Qu.    Max.
##   176.0   553.0   995.0   896.7  1185.0  1450.0
```

```r
summary (temp [ which (temp$AREA == "LT"),  "ELEV" ]) # LT
```

```
##    Min. 1st Qu.  Median    Mean 3rd Qu.    Max.    NA's
##    11.0    89.0   149.0   218.1   229.2  1489.0       2
```

Write sample coordinates to be used with GIS software

Agglomeration beforehand is done only so that the data matches subsequent analyses, just in case agglomerate changes the data (which it shouldn't, but I haven't tested that is doesn't). The `.csv`file is used by in Qgis file `/Zenodo/Qgis/map.qgs` in conjuction with the Quantarctica package. `dplyr` doesn't work here, storing object is necessary.:

```r
coordinates <- sample_data (agglomerate (phsq_ob, "Class"))[ , c ("LONG", "LATI",
  "AREA", "GENE")]
```

See previously defined export path:

```r
write.table (as.data.frame (coordinates), file = path_coord, row.names  = TRUE,
  col.names = TRUE)
```

Garbage collection:

```r
rm (coordinates, temp)
```

Saving intermediate workspace

Saving of temporary work space during coding, so that, code above doesn't have to be run all the time, when changing the tiniest bit downstream.

```
save.image (path_workspace_a)
```

Exploratory data analysis

Isolating data for this analysis

Starting, again by getting the data frames. For now, unless `get_list()` is modified, a lot is crammed into vector `pred_cat` which will be returned in `obs`. Can't include `raw_ages` here, since it's rich in `NA`s and these are filtered by `get_list()`.

```
matr_ana <- get_list (phsq_ob, tax_rank = "Class", pred_cat = c (geochems,
  minerals, position), pres_abs = FALSE)
```

```
## samples -- spc: 88; obs: 88; grp's: 88; gen's: 88
```

Returned as `data.frames` in list are `spc`, `obs`, `grp`, and `gen`.

```
grp <- matr_ana[["grp"]] # for PCA labels
spc <- matr_ana[["spc"]] # species data
```

`obs` is a mixture of things, which need to be treated separately in the following. Hence some isolation work is necessary. The objects are erased from the input list, as a precaution. ** There is a semicolon here!** Afterwards `matr_ana[["obs"]]` is empty.

```
posi <- matr_ana[["obs"]] [ , position]; matr_ana[["obs"]] [ , position] <- NULL
chem <- matr_ana[["obs"]] [ , geochems]; matr_ana[["obs"]] [ , geochems] <- NULL
minl <- matr_ana[["obs"]] [ , minerals]; matr_ana[["obs"]] [ , minerals] <- NULL
```

Checking soil mineral and soil geochemical data

Showing the initial state of the mineral and chemical data

Isolated and combined mineral and chemical data from the `phyloseq` object, congruent with rows in `spc`, `grp`, and `gen`.

```
ggpairs ( data.frame (chem, minl, check.rows = TRUE))
```

```
summary ( data.frame (chem, minl, check.rows = TRUE))
str (data.frame (chem, minl, check.rows = TRUE))
```

Violin plots for unmodified chemical data.

```
add_discretex (chem, grp, dfr_x_name = "AREA") %>%
 get_violinplotlist ( . ,"AREA") %>%
 marrangeGrob ( . , nrow=3, ncol=3)
```

Violin plots for unmodified mineral data.

```
add_discretex (minl, grp, dfr_x_name = "AREA") %>%
 get_violinplotlist ( . ,"AREA") %>%
 marrangeGrob ( . , nrow=3, ncol=3)
```

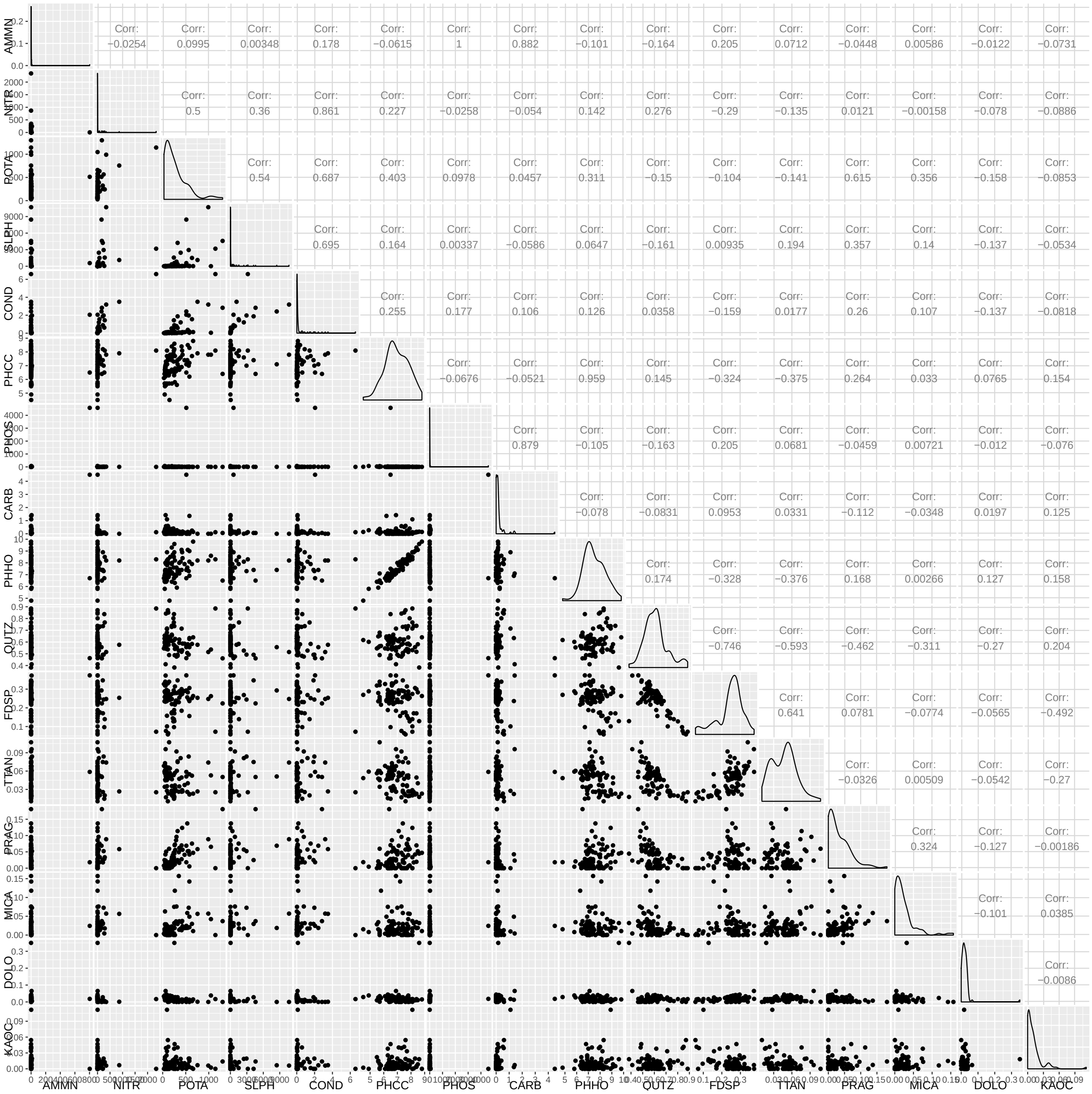

Figure 4: Isolated and combined mineral and chemical data, unmodified. (approx. code line 317)

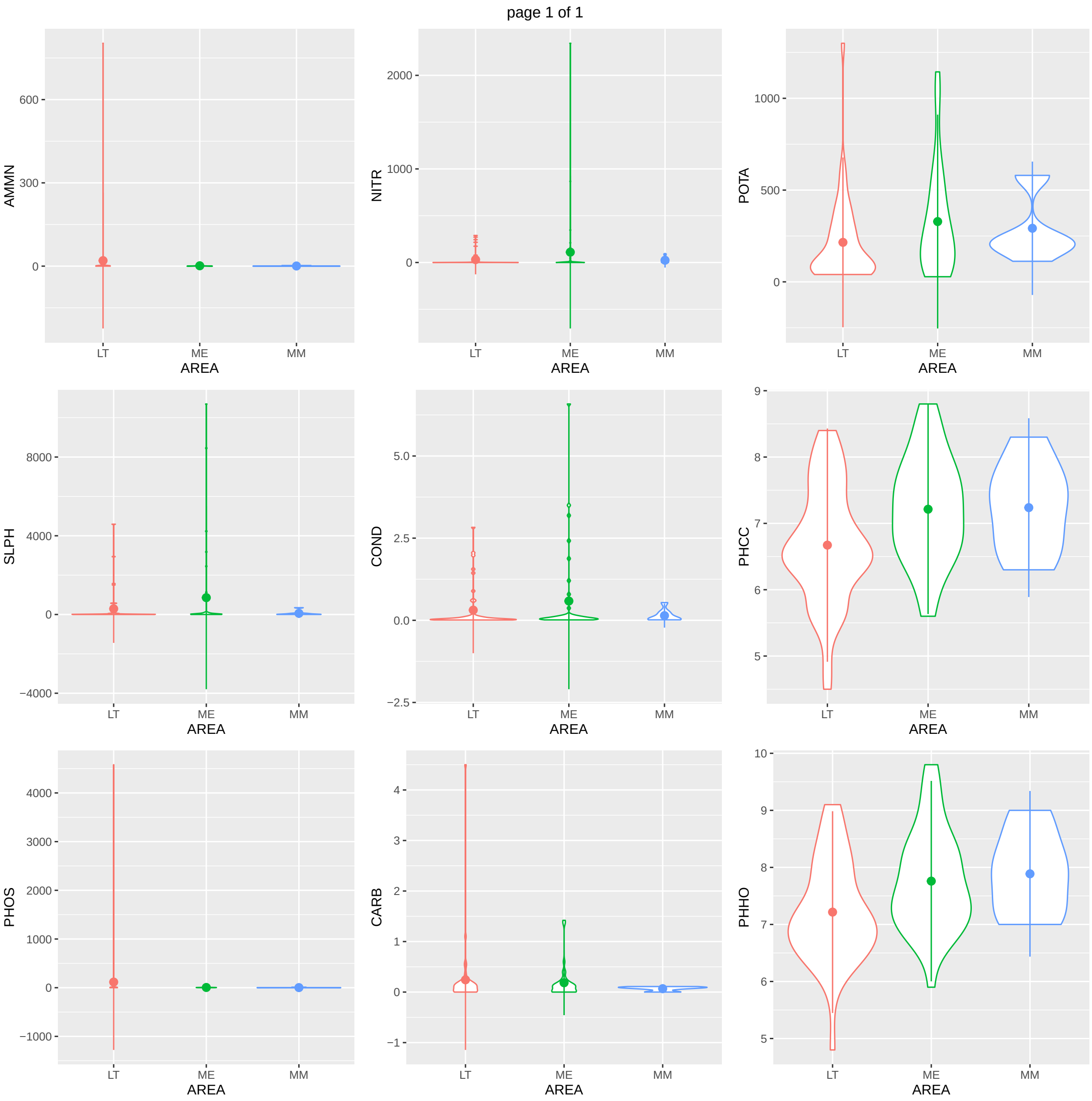

Figure 5: Chemical data per location, unmodified. (approx. code line 323)

Removing outliers in soil geochemical observations

Many ordination approaches are sensitive to outliers, and perhaps this also affects modelling approaches. Outliers are removed here and replaced with the mean. If desired this could perhaps be left out at later stages. This is not done for the rank / compositional mineral data, but only the soil geochemical values.

```
chem <- remove_outliers (chem)
```

```
## This is only adequate for soil geochemical values.
```

```
## There are 9 outliers among 792 values.
```

Transformations

These are helpful for all modelling attempts, PCA, and ANOVA approaches.

Centred log ration transform for the mineral data as recommended by (Ranganathan and Borges 2011), in order to use this data for PCA and alongside the chemical data. The transformation will remove rows from the data farame.

```
minl  <- data.frame (transform_clr (minl))
```

```
## Use this function on compositional data only, such as the mineral
##     data. This function replaces '0' with 'NA's. Adjust the input object via
##     'get_list() if too few samples with complete observations are lost. Check
##     the 'summary()' ouput below to get the 'NA' numbers.
```

```
##       QUTZ             FDSP              TTAN               PRAG
##  Min.   :0.3831   Min.   :0.05801   Min.   :0.01058   Min.   :0.001208
##  1st Qu.:0.5461   1st Qu.:0.22958   1st Qu.:0.02778   1st Qu.:0.012110
##  Median :0.6041   Median :0.25762   Median :0.04884   Median :0.028304
##  Mean   :0.6091   Mean   :0.24564   Mean   :0.04681   Mean   :0.039063
##  3rd Qu.:0.6454   3rd Qu.:0.28747   3rd Qu.:0.05761   3rd Qu.:0.056146
##  Max.   :0.8854   Max.   :0.37358   Max.   :0.10720   Max.   :0.180997
##                                                       NA's   :17
##       MICA               DOLO               KAOC
##  Min.   :0.000294   Min.   :0.000492   Min.   :0.000678
##  1st Qu.:0.008272   1st Qu.:0.010868   1st Qu.:0.003678
##  Median :0.017192   Median :0.018568   Median :0.009219
##  Mean   :0.025778   Mean   :0.024503   Mean   :0.013777
##  3rd Qu.:0.031082   3rd Qu.:0.028240   3rd Qu.:0.015338
##  Max.   :0.157088   Max.   :0.349712   Max.   :0.111665
##  NA's   :9          NA's   :7          NA's   :19
##
##   38 row(s) with NA(s) removed from matrix
##   ** Are the data all in the same measurement units? **
```

Yeo-Johnson transformation conducted here after recombining the data frame (and garbage collection).

```
# merging of data frame and garbage collection
obs <- merge(chem, minl, by = "row.names", all = TRUE); rownames (obs) <-
  obs$Row.names; obs$Row.names <- NULL ; rm (chem); rm(minl)

# transformation of combined mineral an chemical data
obs <- transform_any (obs, method = c ("center", "scale", "YeoJohnson", "nzv"))
```

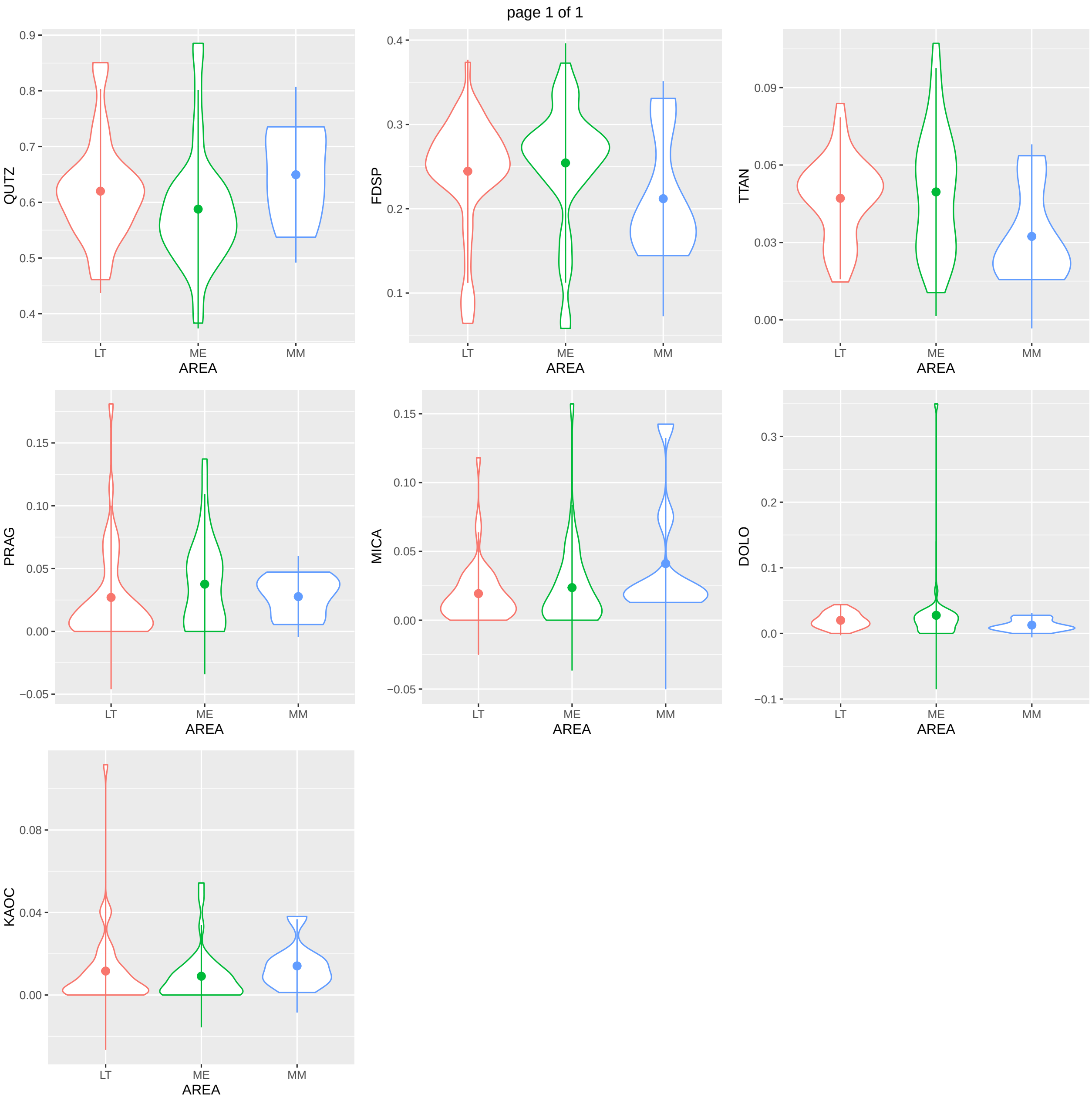

Figure 6: Mineral data per location, unmodified. (approx. code line 329)

```
## Warning in estimateTransform.default(X, Y, weights, family, start,
## method, : Convergence failure: return code = 52
```

```
##       AMMN               NITR               POTA               SLPH        
##  Min.   :-0.8446   Min.   :-0.9396   Min.   :-2.0490   Min.   :-1.9721  
##  1st Qu.:-0.8446   1st Qu.:-0.9396   1st Qu.:-0.9330   1st Qu.:-0.7107  
##  Median :-0.8446   Median :-0.1048   Median : 0.1371   Median :-0.2065  
##  Mean   : 0.0000   Mean   : 0.0000   Mean   : 0.0000   Mean   : 0.0000  
##  3rd Qu.: 1.0885   3rd Qu.: 0.7651   3rd Qu.: 0.7717   3rd Qu.: 0.7605  
##  Max.   : 1.7910   Max.   : 1.7962   Max.   : 2.0561   Max.   : 1.8450  
##                                                                         
##       COND               PHCC               PHOS               CARB         
##  Min.   :-0.8990   Min.   :-2.59105   Min.   :-1.2629   Min.   :-1.18120  
##  1st Qu.:-0.7695   1st Qu.:-0.70087   1st Qu.:-1.2629   1st Qu.:-1.18120  
##  Median :-0.4206   Median :-0.08721   Median : 0.0571   Median : 0.05417  
##  Mean   : 0.0000   Mean   : 0.00000   Mean   : 0.0000   Mean   : 0.00000  
##  3rd Qu.: 0.2853   3rd Qu.: 0.75999   3rd Qu.: 0.7383   3rd Qu.: 0.61452  
##  Max.   : 2.0427   Max.   : 2.18345   Max.   : 2.2058   Max.   : 2.20800  
##                                                                          
##       PHHO               QUTZ               FDSP         
##  Min.   :-2.50966   Min.   :-2.4177   Min.   :-2.81085  
##  1st Qu.:-0.71397   1st Qu.:-0.7710   1st Qu.:-0.56154  
##  Median :-0.06078   Median : 0.1033   Median :-0.07085  
##  Mean   : 0.00000   Mean   : 0.0000   Mean   : 0.00000  
##  3rd Qu.: 0.75269   3rd Qu.: 0.6477   3rd Qu.: 0.53358  
##  Max.   : 2.19578   Max.   : 2.3168   Max.   : 2.10767  
##                     NA's   :38        NA's   :38        
##       TTAN               PRAG               MICA        
##  Min.   :-2.17419   Min.   :-2.38967   Min.   :-2.7368  
##  1st Qu.:-0.75802   1st Qu.:-0.61351   1st Qu.:-0.3565  
##  Median :-0.09433   Median :-0.01572   Median :-0.0634  
##  Mean   : 0.00000   Mean   : 0.00000   Mean   : 0.0000  
##  3rd Qu.: 0.82451   3rd Qu.: 0.62401   3rd Qu.: 0.5225  
##  Max.   : 1.88271   Max.   : 2.49963   Max.   : 2.7383  
##  NA's   :38         NA's   :38         NA's   :38       
##       DOLO               KAOC         
##  Min.   :-2.66544   Min.   :-1.97489  
##  1st Qu.:-0.59736   1st Qu.:-0.90863  
##  Median : 0.08431   Median :-0.01849  
##  Mean   : 0.00000   Mean   : 0.00000  
##  3rd Qu.: 0.63675   3rd Qu.: 0.70536  
##  Max.   : 2.79843   Max.   : 2.06078  
##  NA's   :38         NA's   :38
```

```
obs <- obs[complete.cases(obs), ]
```

Plotting correlations

A simple function call to see precisely, which variables are correlated. Writing correlation plots to disk doesn't work (easily) with `corrplot()` using `ggcorrplot()` instead now (shorten this!).

```
# getting correlations and p-values
corr_all <- ggcorrplot(cor (obs), hc.order = TRUE, type = "lower",
```

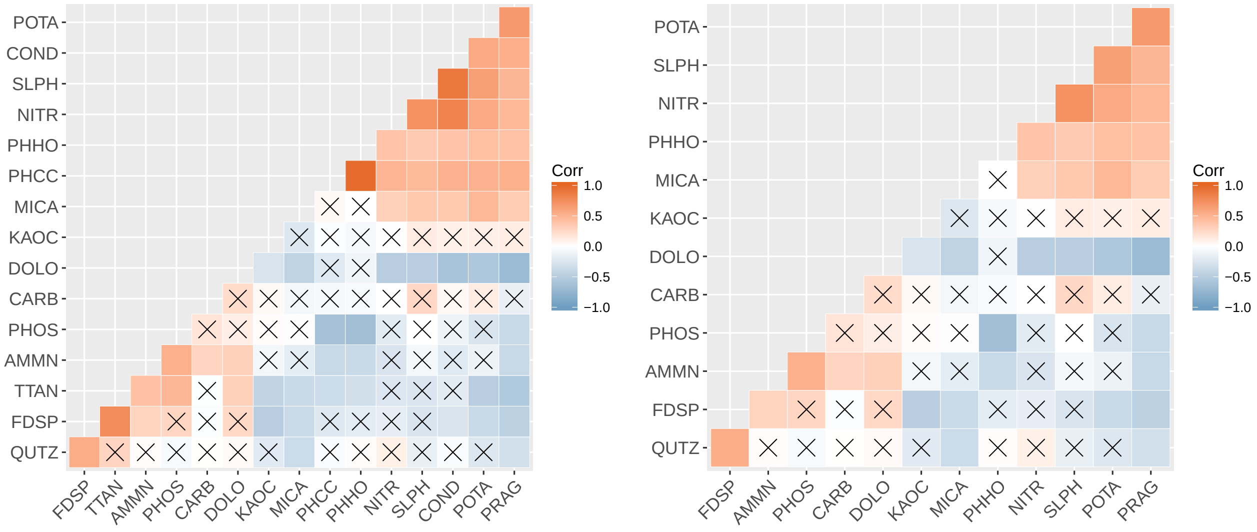

Figure 7: removal of correlations in yj transformed chemical data and clr yj transformed mineral data. (approx. code line 381)

```
  outline.col = "white", p.mat = cor_pmat(obs),
  ggtheme = ggplot2::theme_gray,
  colors = c("#6D9EC1", "white", "#E46726"))

corr_min <-  ggcorrplot(cor (remove_cocorrelated(obs)), hc.order = TRUE, type = "lower",
  outline.col = "white", p.mat = cor_pmat(remove_cocorrelated(obs)),
  ggtheme = ggplot2::theme_gray,
  colors = c("#6D9EC1", "white", "#E46726"))
```

```
## Correlation treshhold is hard-coded with '.75'.

## Above '.75'  (and removed): COND,TTAN,PHCC

## Below '.75' (and retained): AMMN,NITR,POTA,SLPH,PHOS,CARB,PHHO,QUTZ,FDSP,PRAG,MICA,DOLO,KAOC

## Correlation treshhold is hard-coded with '.75'.

## Above '.75'  (and removed): COND,TTAN,PHCC

## Below '.75' (and retained): AMMN,NITR,POTA,SLPH,PHOS,CARB,PHHO,QUTZ,FDSP,PRAG,MICA,DOLO,KAOC
```

```
# plot out
```

```
grid.arrange (corr_all,  corr_min , ncol=2)
```

```
# write to dik
ggsave (path_all_cor, plot = corr_all, dpi = 200, width = 8, height = 4.5,
  units = "in")
ggsave (path_min_cor, plot = corr_min, dpi = 200, width = 8, height = 4.5,
  units = "in")

# garbage cleaning
rm (path_all_cor, path_min_cor, corr_all, corr_min)
```

Removing the correlated variables

In addition to the plot above, the text confirms which variables are kept, and which ones are removed.

```
obs <- remove_cocorrelated(obs)
```

```
## Correlation treshhold is hard-coded with '.75'.
## Above '.75'  (and removed): COND,TTAN,PHCC
## Below '.75' (and retained): AMMN,NITR,POTA,SLPH,PHOS,CARB,PHHO,QUTZ,FDSP,PRAG,MICA,DOLO,KAOC
```

Plotting out transformed mineral and chemical data

Plot the data before it goes to into further analyses, only for checking. Initially a scatterplot.

```
# saving this plot for disk-write
ggpairs (obs) # less correlation, less skew?
```

```
summary (obs)

# write data to disk (slooooooow)
pdf(path_sca_tra, height = 10, width = 10)
g <- ggpairs(obs)
print(g)
dev.off()

# garbage collection
rm (g)
```

And the violin plots.

```
add_discretex (obs, grp, dfr_x_name = "AREA") %>%
 get_violinplotlist ( . ,"AREA") %>%
 marrangeGrob ( . , nrow=5, ncol=3)
```

```
# write data to disk (slooooooow)
g <- add_discretex (obs, grp, dfr_x_name = "AREA") %>%
 get_violinplotlist ( . ,"AREA") %>%
 marrangeGrob ( . , nrow=5, ncol=3)

ggsave (path_vio_tra, plot = g, dpi = 200, width = 9, height = 9,
  units = "in")

# garbage collection
rm (g)
```

Principal component analysis of observations

Getting the principal components

Principal components are retrieved for the `obs` data frame with the mineral analysis values.

```
pcs <- prcomp (obs, center = FALSE, scale = FALSE)
```

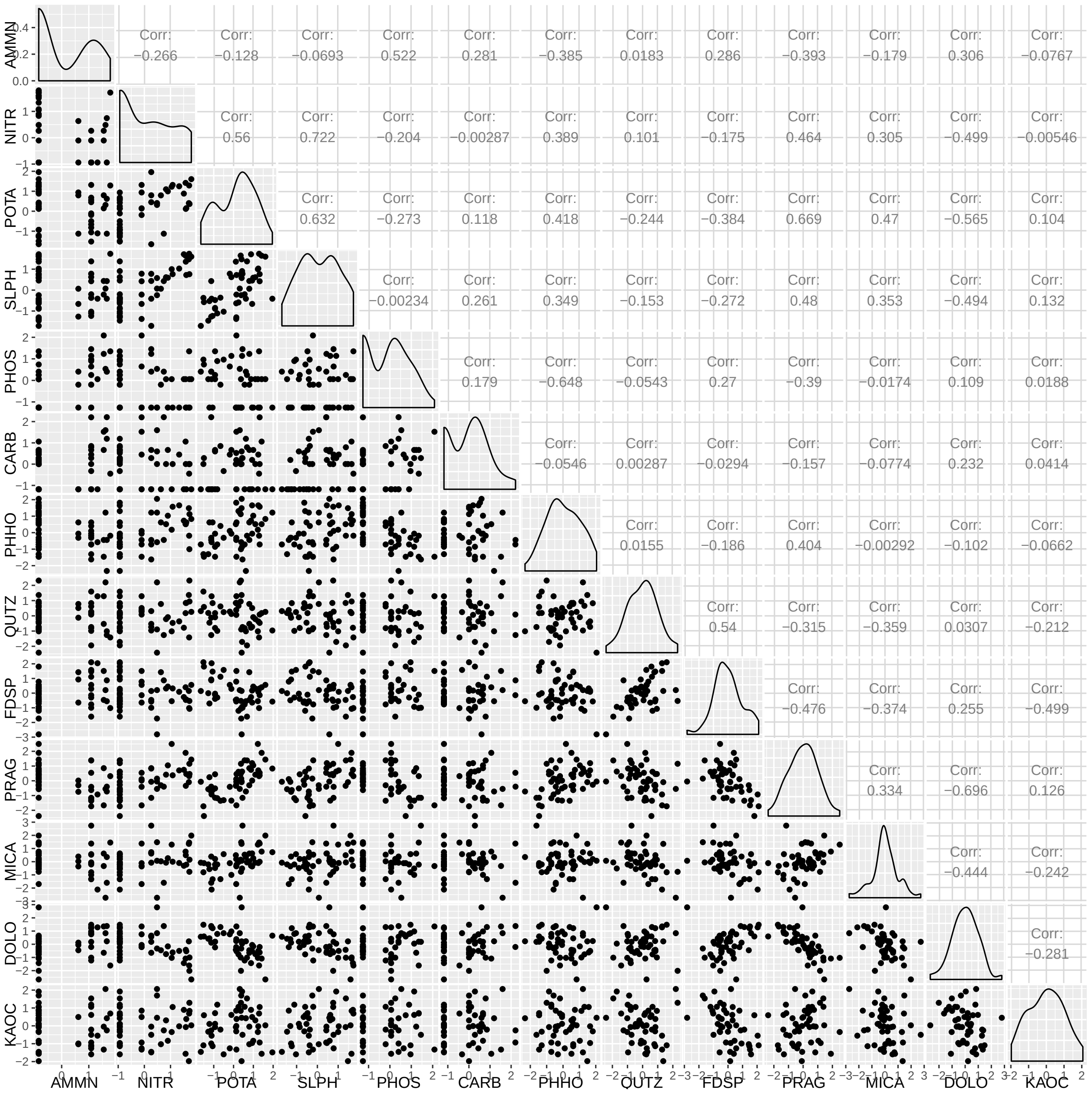

Figure 8: Scatterplot of centred and scaled mineral and chemical data (approx. code line 404).

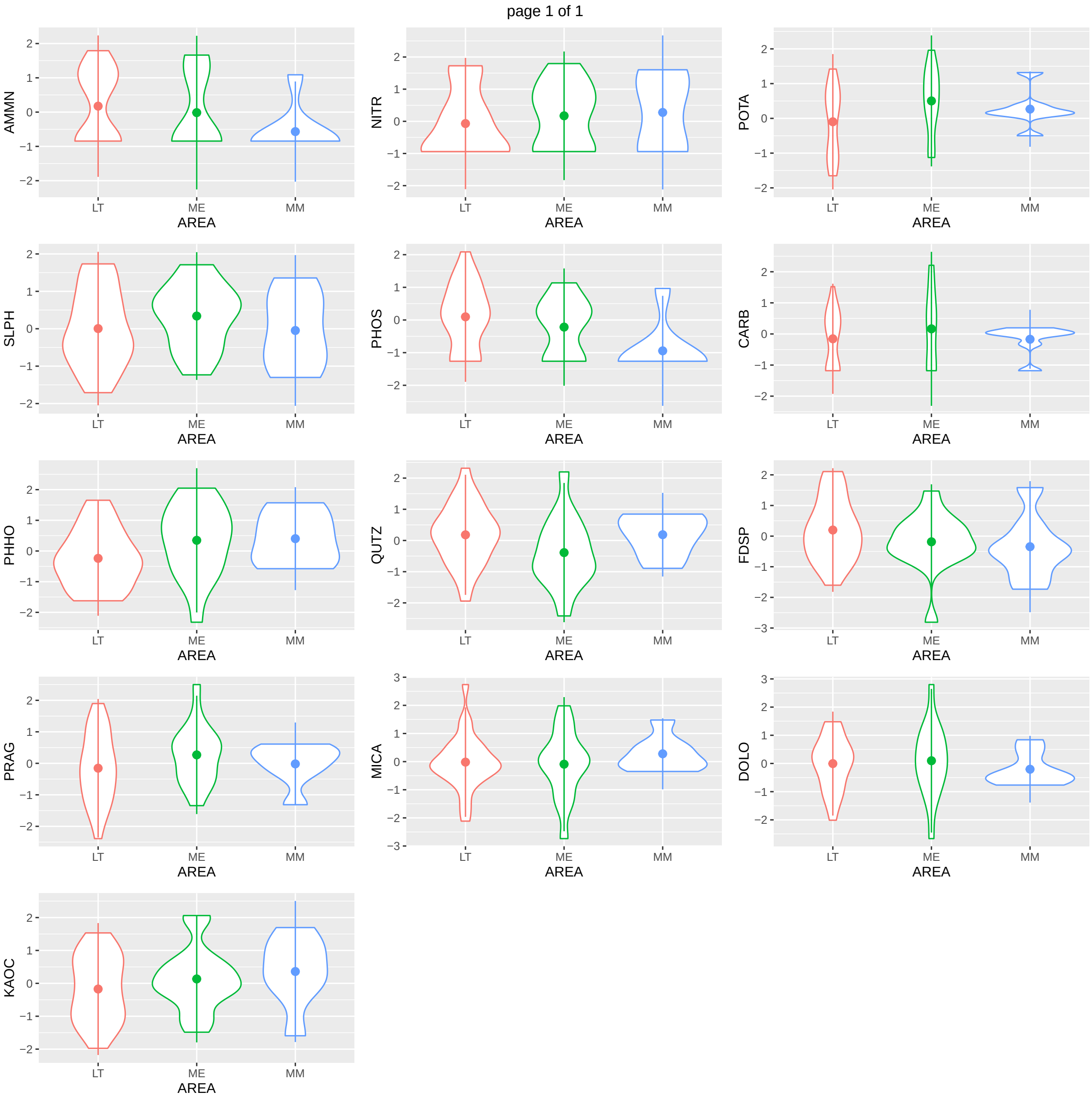

Figure 9: Violin plot of scaled mineral and chemical data. Note that several samples have been excluded when transforming merging mineral and chemical data (approx. code line 419).

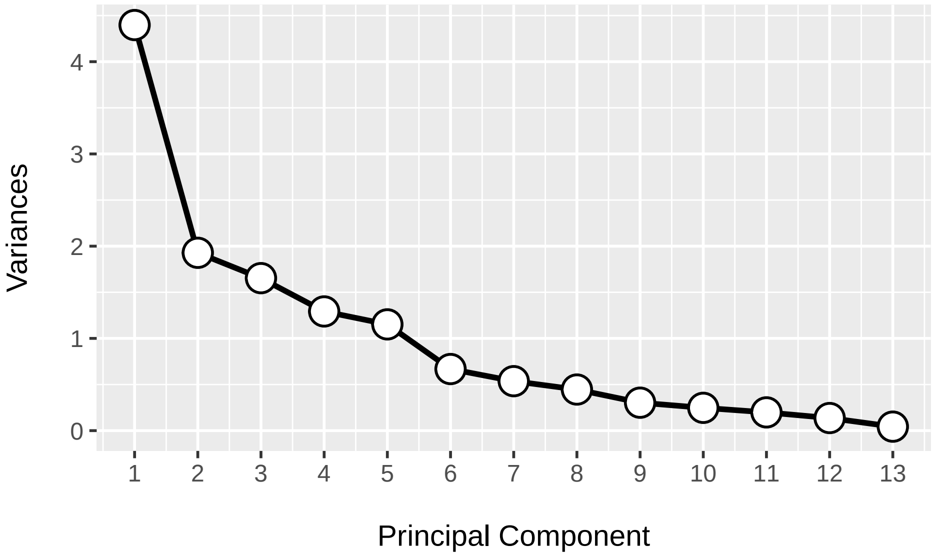

Figure 10: PCA variance plots of centred and scaled mineral data (approx. code line 452)

Testing the principal components

Testing the principal components with a variance plot. Here on should only interpret principal components with variances above 1.

```
plot_pcvars(pcs)
```

```
# write to disk
ggsave (path_pca_var, plot = last_plot (), path = NULL, scale = 1, width = 7,
  height = 3, units = "in", dpi = 300)
```

Bi-plot

The biplot can be generated, after the variables in `grp` are subset to match the `pcs` object, necessary for correct ovals in the plot.

```
get_biplot(pcs, shorten_groups (grp, obs)) # `grp` is needed later without type
```

```
                                           #  conversion - don't expand this
                                           #  expression!

# write to disk
ggsave (path_pca_bip, plot = last_plot (), scale = 1, width = 7,
  height = 7, units = "in", dpi = 300)
```

Checking species information

Showing the initial state of the species information

Here used is the initial abundance data, which is abundance corrected using the CSS algorithm in Qiime (Paulson et al. 2013) *"With CSS, raw counts are divided by the cumulative sum of counts up to a percentile determined using a data-driven approach."* The data is shown using a scatterplot and a violin plot.

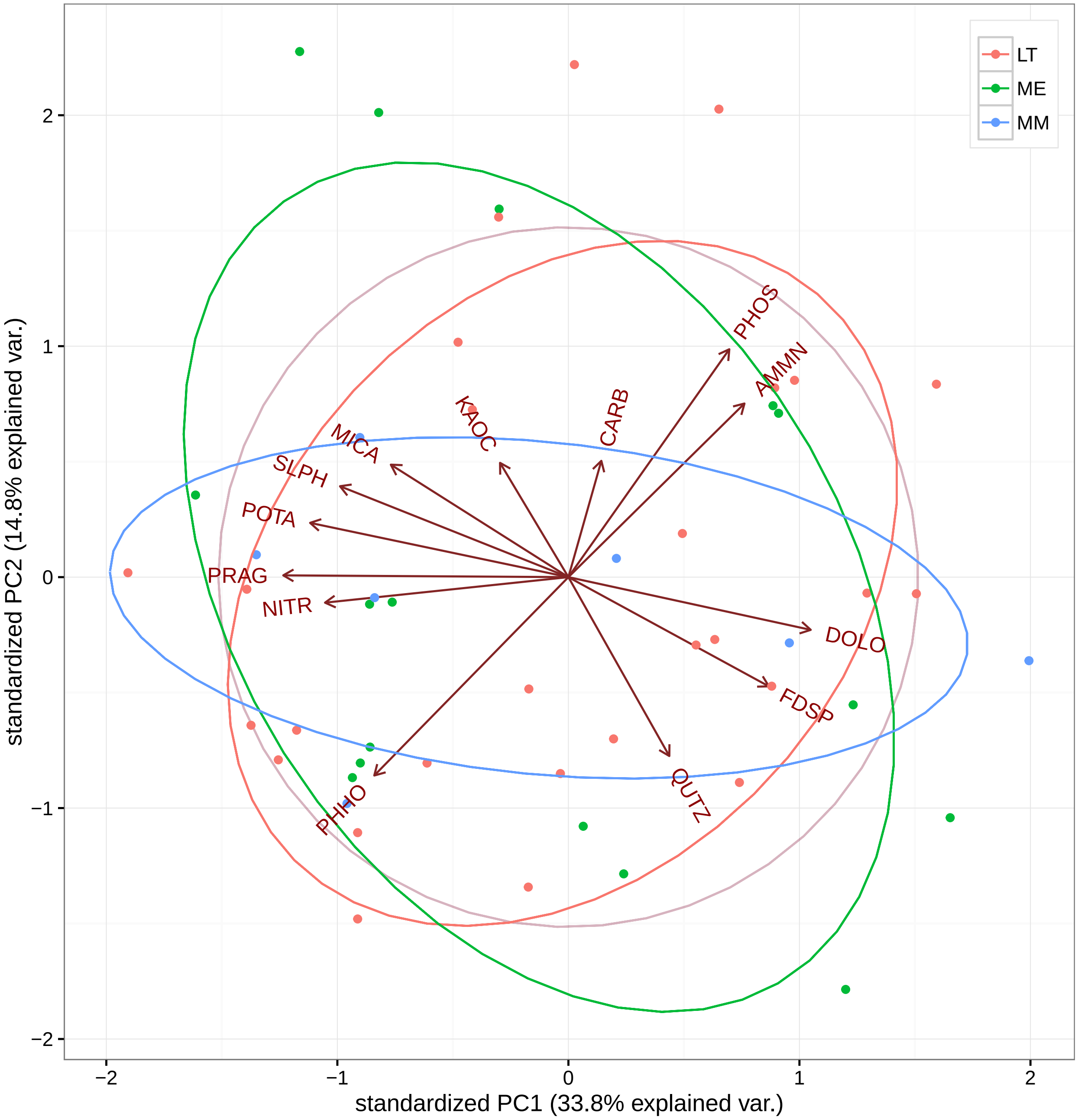

Figure 11: PCA biplots on clr/vj-transformed mineral data and yj-transformed chemical data. (approx. code line 463)

```r
ggpairs (spc)
```

```r
summary (spc)
```

```r
add_discretex ( as.data.frame(spc), grp, dfr_x_name = "AREA") %>%
 get_violinplotlist ( . ,"AREA") %>%
 marrangeGrob ( . , nrow=3, ncol=2)
```

Non-Metric Multidimensional Scaling

Matching up phylotype and factor information.

Species observations have to match the observations.

```r
spc <- cmpl_phylotypes(spc, obs)
```

Getting a `metaMDS` object from the species data

Calculating a `metaMDS` object on the `spc` data, for now without environmental variables. Distance `jaccard` appears to be recommended in `vegan()`, `binary = TRUE` ensures correct distance calculation on presence / absence data. `try` is set higher, to look longer for solutions. Stress > 0.05 provides an excellent representation in reduced dimensions, > 0.1 is great, > 0.2 is fair, and stress > 0.3 provides a poor representation. Inspect the generated model in the end.

```r
spc_mds <- metaMDS(spc, distance = "bray" ,  k = 2, try = 1000, trymax = 2000,
  noshare = FALSE, wascores = TRUE, trace = 0, plot = FALSE, expand = TRUE,
  binary = FALSE)

spc_mds # stress value is 0.04411049
```

```
##
## Call:
## metaMDS(comm = spc, distance = "bray", k = 2, try = 1000, trymax = 2000,      noshare = FALSE, wasco
##
## global Multidimensional Scaling using monoMDS
##
## Data:     wisconsin(sqrt(spc))
## Distance: bray
##
## Dimensions: 2
## Stress:     0.04411049
## Stress type 1, weak ties
## Two convergent solutions found after 1000 tries
## Scaling: centring, PC rotation, halfchange scaling
## Species: expanded scores based on 'wisconsin(sqrt(spc))'
```

```r
stressplot(spc_mds)
```

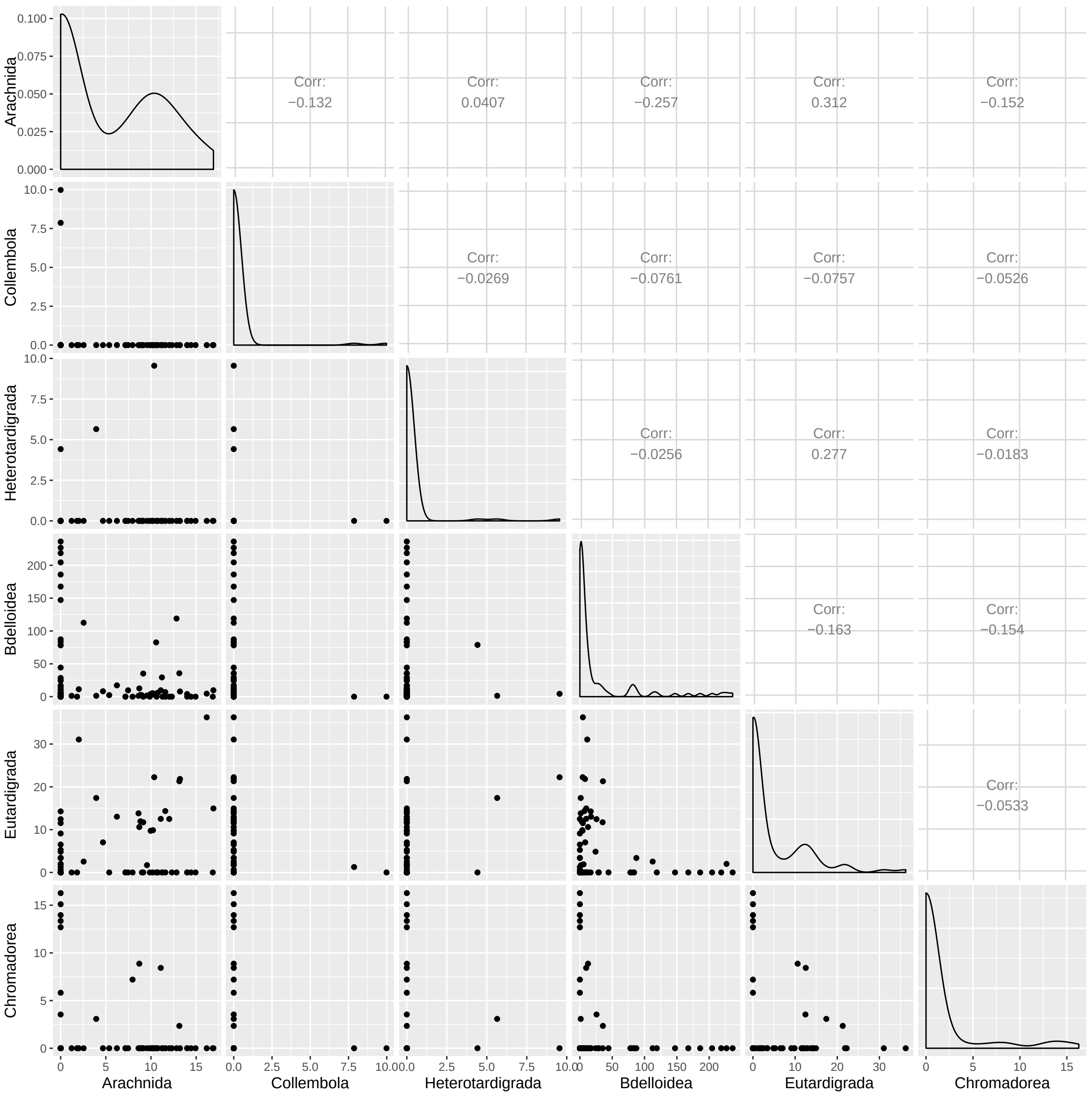

Figure 12: Scatterplot of cumulative sum-scaled species observations (approx. code line 485).

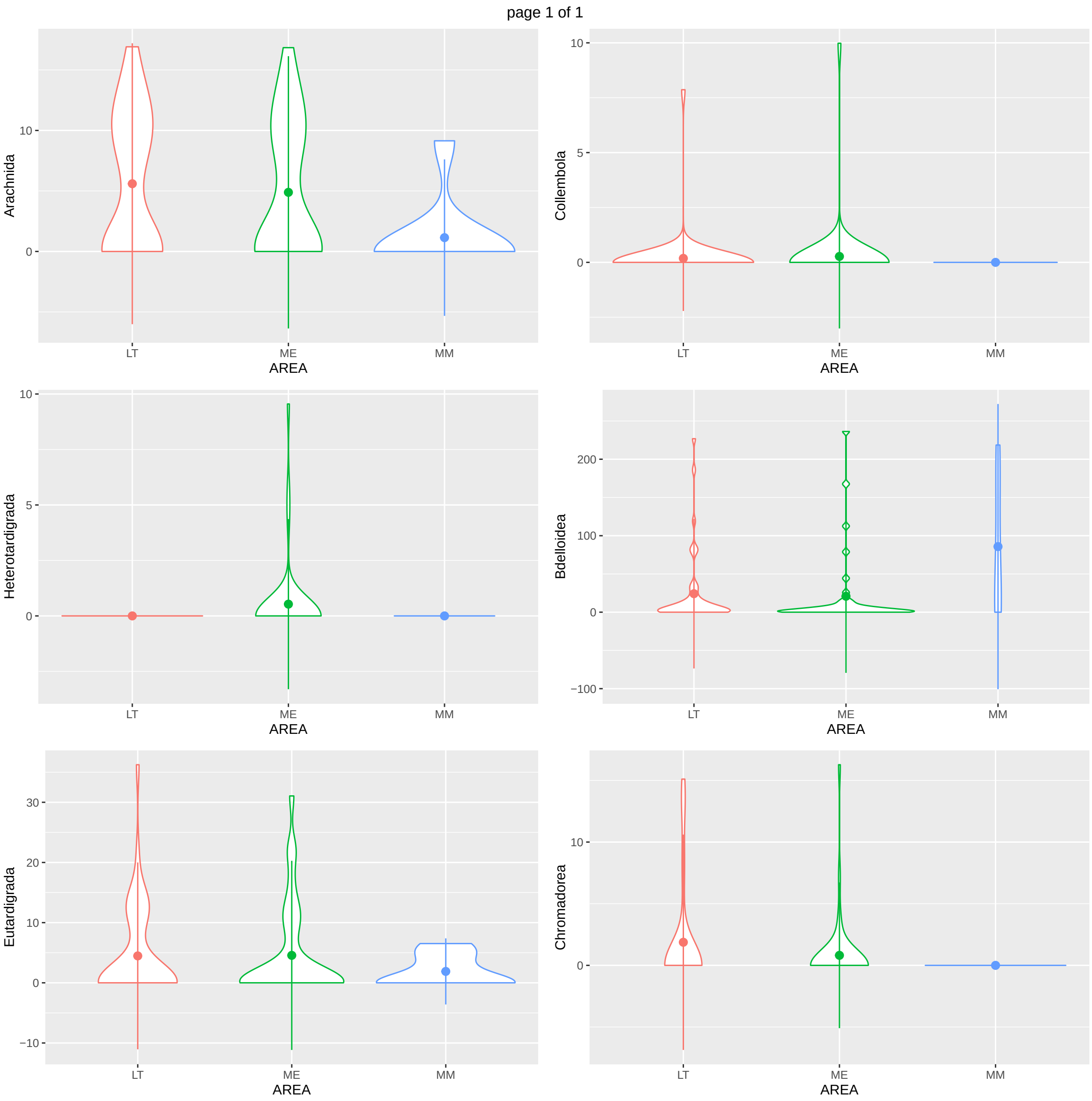

Figure 13: Violin plot of cumulative sum-scaled species observations (approx. code line 488).

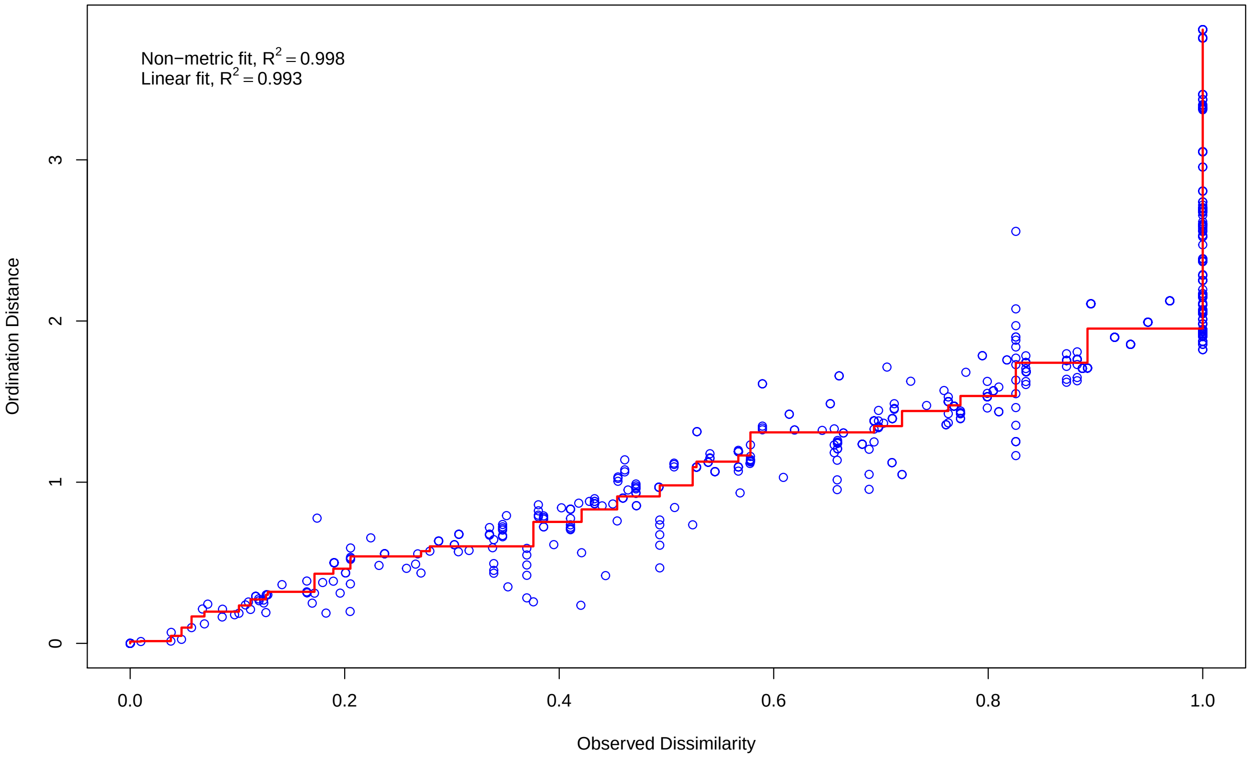

Figure 14: NMDS stressplot (approx. code line 519).

Fitting environmental vectors

```
env <- envfit(spc_mds, obs, permutations = 9999)
env
```

```
##
## ***VECTORS
##
##          NMDS1    NMDS2     r2 Pr(>r)
## AMMN  0.94781  0.31884 0.0349 0.4383
## NITR -0.96888  0.24755 0.0647 0.2091
## POTA -0.98747  0.15779 0.0563 0.2610
## SLPH -0.99542 -0.09561 0.1671 0.0126 *
## PHOS -0.09385  0.99559 0.0288 0.5109
## CARB -0.96239  0.27168 0.0189 0.6364
## PHHO  0.16724 -0.98592 0.0069 0.8444
## QUTZ  0.18587  0.98257 0.0225 0.5857
## FDSP  0.35795  0.93374 0.0867 0.1180
## PRAG -0.77887 -0.62719 0.0813 0.1374
## MICA -0.99957  0.02938 0.0196 0.6272
## DOLO  0.51791 -0.85543 0.0755 0.1601
## KAOC  0.93373 -0.35797 0.0008 0.9785
## ---
## Signif. codes:  0 '***' 0.001 '**' 0.01 '*' 0.05 '.' 0.1 ' ' 1
## Permutation: free
## Number of permutations: 9999
```

```r
par (mfrow = c (1, 1))
ordiplot (spc_mds, display = "sites" )
# ordihull (spc_mds, shorten_groups (grp, obs), col = c ("coral3", "chartreuse4", "cornflowerblue"))
ordiellipse(spc_mds, shorten_groups (grp, obs) , display = "sites", kind = "se",
  conf = 0.95, label = T,  col = c ("red", "chartreuse4", "cornflowerblue"))
orditorp (spc_mds, display = "species", col="deepskyblue4", air = 0.01, cex = 1.0)
with (obs, ordisurf (spc_mds, SLPH, add = TRUE, col = "darkgoldenrod4" ))
```

```
##
## Family: gaussian
## Link function: identity
##
## Formula:
## y ~ s(x1, x2, k = 10, bs = "tp", fx = FALSE)
##
## Estimated degrees of freedom:
## 2.56  total = 3.56
##
## REML score: 67.78314
```

```r
# with (obs, ordisurf (spc_mds, AMMN, add = TRUE, col = "orange"))

# write data to disk (sloooooooow)
pdf(path_mds_hip, height = 9, width = 9)
par (mfrow = c (1, 1))
ordiplot (spc_mds, display = "sites" )
# ordihull (spc_mds, shorten_groups (grp, obs), col = c ("coral3", "chartreuse4", "cornflowerblue"))
ordiellipse(spc_mds, shorten_groups (grp, obs) , display = "sites", kind = "se",
  conf = 0.95, label = T,  col = c ("red", "chartreuse4", "cornflowerblue"))
orditorp (spc_mds, display = "species", col="deepskyblue4", air = 0.01, cex = 1.0)
with (obs, ordisurf (spc_mds, SLPH, add = TRUE, col = "darkgoldenrod4" ))
```

```
##
## Family: gaussian
## Link function: identity
##
## Formula:
## y ~ s(x1, x2, k = 10, bs = "tp", fx = FALSE)
##
## Estimated degrees of freedom:
## 2.56  total = 3.56
##
## REML score: 67.78314
```

```r
# with (obs, ordisurf (spc_mds, AMMN, add = TRUE, col = "orange"))
dev.off()
```

```
## pdf
##   2
```

adonis analysis

Class beta diversity, expressed as distance `z = (log(2)-log(2*a+b+c)+log(a+b+c))/log(2)` may be function of group means of SLPH.

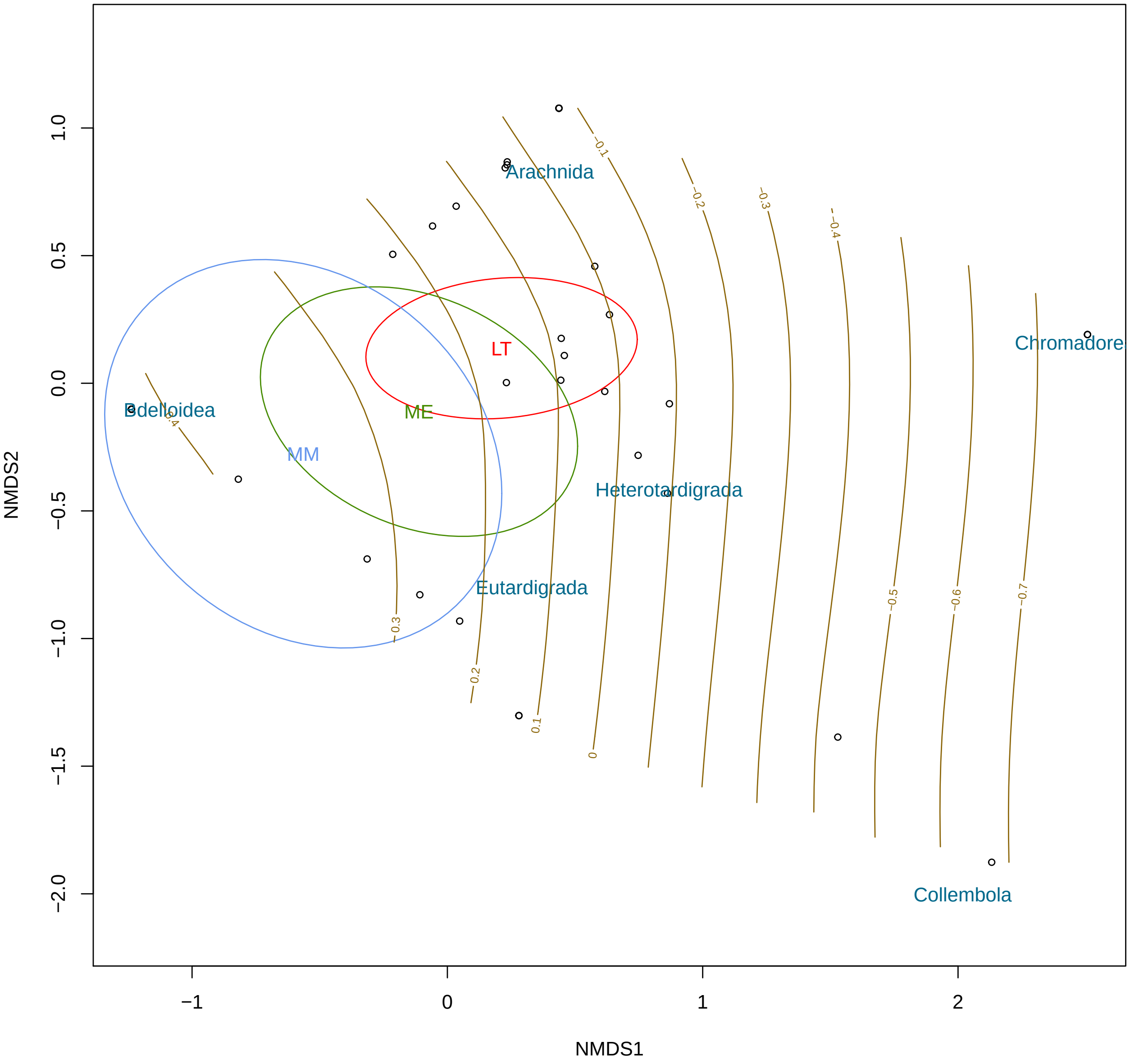

Figure 15: NMDS plot of species and mineral data, SLPH with (*) significance here. Blue: Mount Menzies, Red: Lake Terrasovoje, Green: Mawson Escarpment (approx. code line 527).

```r
ad <- adonis (formula =  betadiver ( spc, "z") ~  SLPH, data = obs, perm = 9999)
ad
```

```
##
## Call:
## adonis(formula = betadiver(spc, "z") ~ SLPH, data = obs, permutations = 9999)
##
## Permutation: free
## Number of permutations: 9999
##
## Terms added sequentially (first to last)
##
##           Df SumsOfSqs MeanSqs F.Model      R2 Pr(>F)
## SLPH       1    0.9859 0.98591  4.3985 0.08394 0.0074 **
## Residuals 48   10.7591 0.22415         0.91606
## Total     49   11.7450                 1.00000
## ---
## Signif. codes:  0 '***' 0.001 '**' 0.01 '*' 0.05 '.' 0.1 ' ' 1
```

Canonical correspondence analysis

Calculate the CCA

Here using also the Sulphur variables, as it was the only one that showed persistent significance across all tests. Show the result.

```r
inv_slph <- cca(spc ~ SLPH , data = obs)
inv_slph
```

```
## Call: cca(formula = spc ~ SLPH, data = obs)
##
##               Inertia Proportion Rank
## Total         2.97792    1.00000
## Constrained   0.12347    0.04146    1
## Unconstrained 2.85445    0.95854    5
## Inertia is mean squared contingency coefficient
##
## Eigenvalues for constrained axes:
##    CCA1
## 0.12347
##
## Eigenvalues for unconstrained axes:
##    CA1    CA2    CA3    CA4    CA5
## 0.9214 0.7444 0.6849 0.3642 0.1395
```

```r
# output of the summary function is very long, I'll mute this
# summary(inv_slph)
```

Testing CCA results

Permutation testing

```r
anova (inv_slph, perm = 9999)
```

```
## Permutation test for cca under reduced model
## Permutation: free
## Number of permutations: 999
##
## Model: cca(formula = spc ~ SLPH, data = obs)
##          Df ChiSquare      F Pr(>F)
## Model     1   0.12347 2.0762  0.047 *
## Residual 48   2.85445
## ---
## Signif. codes:  0 '***' 0.001 '**' 0.01 '*' 0.05 '.' 0.1 ' ' 1
```

Testing the (single) axis for significance

```
anova (inv_slph, by="axis", perm = 1000)
```

```
## Permutation test for cca under reduced model
## Marginal tests for axes
## Permutation: free
## Number of permutations: 999
##
## Model: cca(formula = spc ~ SLPH, data = obs)
##          Df ChiSquare      F Pr(>F)
## CCA1      1   0.12347 2.0762  0.033 *
## Residual 48   2.85445
## ---
## Signif. codes:  0 '***' 0.001 '**' 0.01 '*' 0.05 '.' 0.1 ' ' 1
```

Type I test

```
anova (inv_slph, by="term", perm = 1000) # SLPH
```

```
## Permutation test for cca under reduced model
## Terms added sequentially (first to last)
## Permutation: free
## Number of permutations: 999
##
## Model: cca(formula = spc ~ SLPH, data = obs)
##          Df ChiSquare      F Pr(>F)
## SLPH      1   0.12347 2.0762  0.042 *
## Residual 48   2.85445
## ---
## Signif. codes:  0 '***' 0.001 '**' 0.01 '*' 0.05 '.' 0.1 ' ' 1
```

Type III test

```
anova(inv_slph, by="margin", perm = 1000) # SLPH
```

```
## Permutation test for cca under NA model
## Marginal effects of terms
## Permutation: free
## Number of permutations: 999
##
## Model: cca(formula = spc ~ SLPH, data = obs)
##          Df ChiSquare      F Pr(>F)
## SLPH      1   0.12347 2.0762  0.035 *
## Residual 48   2.85445
## ---
## Signif. codes:  0 '***' 0.001 '**' 0.01 '*' 0.05 '.' 0.1 ' ' 1
```

Variance inflation factor

```
vif.cca(inv_slph)
```

```
## SLPH
##    1
```

Goodness of fit for classes

```
goodness(inv_slph)
```

```
##                          CCA1
## Arachnida        0.024956380
## Collembola       0.032179458
## Heterotardigrada 0.003604218
## Bdelloidea       0.090319003
## Eutardigrada     0.009349762
## Chromadorea      0.077083565
```

Regression analyses

Across all samples

Isolate ages for analysis, get_list() prunes undefined values across the whole returned table.

```
ages <- get_list (phsq_ob, tax_rank = "Class", pred_cat = c (raw_ages, geochems,
  minerals), pres_abs = FALSE)
```

```
## samples -- spc: 32; obs: 32; grp's: 32; gen's: 32
```

Extract observations from list into dataframe to work with them more easily.

```
ages <- ages[["obs"]];
```

Get the row means across higher and lower estimates, only one column is desired for further analysis.

```
ages$MNAGE <- -1 * rowMeans (ages[ ,c("HAGE", "LAGE")])
```

Plot all regressions

Make plotting easier by using `lapply()` to generate plots, for which one needs a vector to loop over.

```
y_axis <- c("AMMN", "NITR", "POTA", "SLPH", "COND", "PHCC", "PHOS", "CARB",
               "PHHO")
```

Create a list of graphical objects for plotting on one page.

```
regr_list <- lapply (y_axis, plot_regrs, ages)
```

Plots can now be shown, and saved to the output directory. Objects are then discarded.

```
marrangeGrob (regr_list, nrow = 3, ncol = 3)
```

Saving plots.:

```
ggsave (file = path_regr, plot = marrangeGrob (regr_list, nrow = 3, ncol = 3),
        dpi = 200, width = 10, height = 10, units = "in")
```

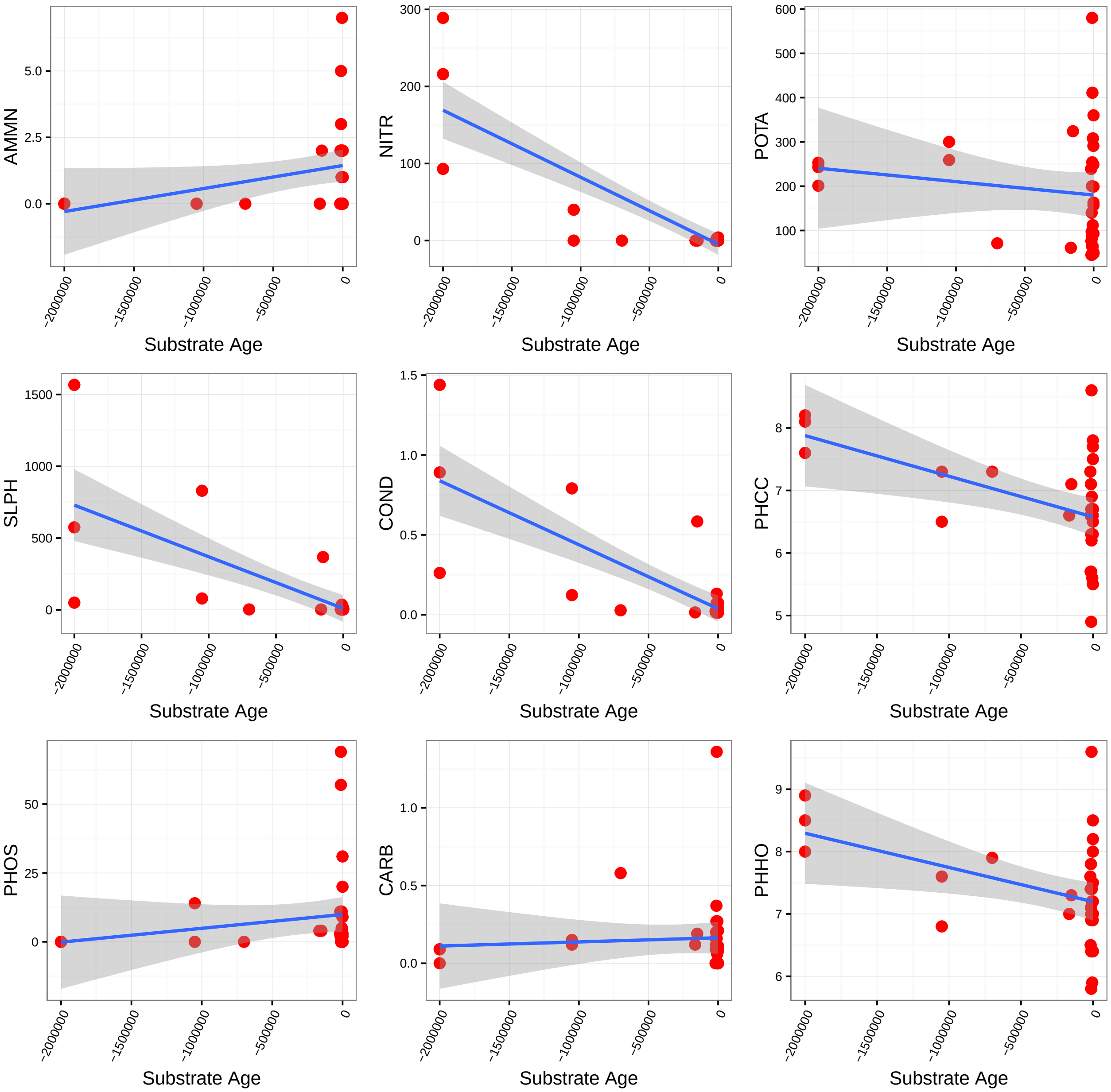

Figure 16: Regressions between all terrrain ages and soil geochemical measurments. (Approx. code line 635)

Testing all regressions

Calculate all regressions, with variables from previous section, and store as list.

```
regressions <- lapply (y_axis, function(x) {
    lm ( substitute (MNAGE ~ i, list(i = as.name(x))), data = ages)
    })
```

Return summary from list for more specific results

```
lapply (regressions, summary )
```

```
## [[1]]
##
## Call:
## lm(formula = substitute(MNAGE ~ i, list(i = as.name(x))), data = ages)
##
## Residuals:
##      Min       1Q   Median       3Q      Max
## -1552321   -31959   178851   343493   447179
##
## Coefficients:
##             Estimate Std. Error t value Pr(>|t|)
## (Intercept)  -447679     132154  -3.388  0.00199 **
## AMMN          132414      67134   1.972  0.05785 .
## ---
## Signif. codes:  0 '***' 0.001 '**' 0.01 '*' 0.05 '.' 0.1 ' ' 1
##
## Residual standard error: 596200 on 30 degrees of freedom
## Multiple R-squared:  0.1148, Adjusted R-squared:  0.08528
## F-statistic:  3.89 on 1 and 30 DF,  p-value: 0.05785
##
##
## [[2]]
##
## Call:
## lm(formula = substitute(MNAGE ~ i, list(i = as.name(x))), data = ages)
##
## Residuals:
##      Min       1Q   Median       3Q      Max
## -1111688   104883   114883   120883   504636
##
## Coefficients:
##              Estimate Std. Error t value Pr(>|t|)
## (Intercept) -121383.3    62665.4  -1.937   0.0622 .
## NITR          -8246.5      945.8  -8.719 1.01e-09 ***
## ---
## Signif. codes:  0 '***' 0.001 '**' 0.01 '*' 0.05 '.' 0.1 ' ' 1
##
## Residual standard error: 337100 on 30 degrees of freedom
## Multiple R-squared:  0.717,  Adjusted R-squared:  0.7076
## F-statistic: 76.02 on 1 and 30 DF,  p-value: 1.006e-09
##
##
## [[3]]
##
```

```
## Call:
## lm(formula = substitute(MNAGE ~ i, list(i = as.name(x))), data = ages)
##
## Residuals:
##      Min       1Q   Median       3Q      Max
## -1700721   173102   215127   300208   564243
##
## Coefficients:
##              Estimate Std. Error t value Pr(>|t|)
## (Intercept) -153454.7   200333.1  -0.766    0.450
## POTA           -725.5      884.0  -0.821    0.418
##
## Residual standard error: 626700 on 30 degrees of freedom
## Multiple R-squared:  0.02196,    Adjusted R-squared:  -0.01064
## F-statistic: 0.6735 on 1 and 30 DF,  p-value: 0.4183
##
##
## [[4]]
##
## Call:
## lm(formula = substitute(MNAGE ~ i, list(i = as.name(x))), data = ages)
##
## Residuals:
##      Min       1Q   Median       3Q      Max
## -1796795   126451   134690   146254   482782
##
## Coefficients:
##              Estimate Std. Error t value Pr(>|t|)
## (Intercept) -135534.0    85351.2  -1.588    0.123
## SLPH          -1353.4      253.7  -5.334 9.08e-06 ***
## ---
## Signif. codes:  0 '***' 0.001 '**' 0.01 '*' 0.05 '.' 0.1 ' ' 1
##
## Residual standard error: 454000 on 30 degrees of freedom
## Multiple R-squared:  0.4867, Adjusted R-squared:  0.4696
## F-statistic: 28.45 on 1 and 30 DF,  p-value: 9.078e-06
##
##
## [[5]]
##
## Call:
## lm(formula = substitute(MNAGE ~ i, list(i = as.name(x))), data = ages)
##
## Residuals:
##      Min       1Q   Median       3Q      Max
## -1547053    68042    79948   116116   788591
##
## Coefficients:
##             Estimate Std. Error t value Pr(>|t|)
## (Intercept)   -57796      78431  -0.737    0.467
## COND        -1508210     222937  -6.765 1.68e-07 ***
## ---
## Signif. codes:  0 '***' 0.001 '**' 0.01 '*' 0.05 '.' 0.1 ' ' 1
##
```

```
## Residual standard error: 398800 on 30 degrees of freedom
## Multiple R-squared:  0.6041, Adjusted R-squared:  0.5909
## F-statistic: 45.77 on 1 and 30 DF,  p-value: 1.68e-07
##
##
## [[6]]
##
## Call:
## lm(formula = substitute(MNAGE ~ i, list(i = as.name(x))), data = ages)
##
## Residuals:
##      Min       1Q   Median       3Q      Max
## -1417583  -130083   161491   266973   922355
##
## Coefficients:
##             Estimate Std. Error t value Pr(>|t|)
## (Intercept)  2077113     802809   2.587  0.01476 *
## PHCC         -349938     117764  -2.972  0.00579 **
## ---
## Signif. codes:  0 '***' 0.001 '**' 0.01 '*' 0.05 '.' 0.1 ' ' 1
##
## Residual standard error: 557000 on 30 degrees of freedom
## Multiple R-squared:  0.2274, Adjusted R-squared:  0.2016
## F-statistic:  8.83 on 1 and 30 DF,  p-value: 0.005789
##
##
## [[7]]
##
## Call:
## lm(formula = substitute(MNAGE ~ i, list(i = as.name(x))), data = ages)
##
## Residuals:
##      Min       1Q   Median       3Q      Max
## -1644087    62608   298293   333902   355413
##
## Coefficients:
##             Estimate Std. Error t value Pr(>|t|)
## (Intercept)  -355913     124882  -2.850  0.00783 **
## PHOS            7732       7019   1.101  0.27945
## ---
## Signif. codes:  0 '***' 0.001 '**' 0.01 '*' 0.05 '.' 0.1 ' ' 1
##
## Residual standard error: 621300 on 30 degrees of freedom
## Multiple R-squared:  0.03887,    Adjusted R-squared:  0.006831
## F-statistic: 1.213 on 1 and 30 DF,  p-value: 0.2795
##
##
## [[8]]
##
## Call:
## lm(formula = substitute(MNAGE ~ i, list(i = as.name(x))), data = ages)
##
## Residuals:
##      Min       1Q   Median       3Q      Max
```

```
## -1698886   134069   281777   302574   315119
##
## Coefficients:
##             Estimate Std. Error t value Pr(>|t|)
## (Intercept)  -315619     131904  -2.393   0.0232 *
## CARB          161162     448129   0.360   0.7216
## ---
## Signif. codes:  0 '***' 0.001 '**' 0.01 '*' 0.05 '.' 0.1 ' ' 1
##
## Residual standard error: 632300 on 30 degrees of freedom
## Multiple R-squared:  0.004293,	Adjusted R-squared:  -0.0289
## F-statistic: 0.1293 on 1 and 30 DF,  p-value: 0.7216
##
##
## [[9]]
##
## Call:
## lm(formula = substitute(MNAGE ~ i, list(i = as.name(x))), data = ages)
##
## Residuals:
##      Min       1Q   Median       3Q      Max
## -1505945   -61811   155191   290151   990134
##
## Coefficients:
##             Estimate Std. Error t value Pr(>|t|)
## (Intercept)  2036340     932610   2.183   0.0370 *
## PHHO         -316299     126020  -2.510   0.0177 *
## ---
## Signif. codes:  0 '***' 0.001 '**' 0.01 '*' 0.05 '.' 0.1 ' ' 1
##
## Residual standard error: 576100 on 30 degrees of freedom
## Multiple R-squared:  0.1735,	Adjusted R-squared:  0.146
## F-statistic:   6.3 on 1 and 30 DF,  p-value: 0.0177
```

Region specific

Isolate ages for analysis, get_list() prunes undefined values across the whole returned table.

```
ages <- get_list (phsq_ob, tax_rank = "Class", pred_cat = c (raw_ages, geochems,
  minerals), pres_abs = FALSE)
```

```
## samples -- spc: 32; obs: 32; grp's: 32; gen's: 32
```

Extract observations from list into dataframe to work with them more easily.

```
ages <- cbind(ages[["obs"]], ages[["grp"]])
```

Get the row means across higher and lower estimates, only one column is desired for further analysis.

```
ages$MNAGE <- -1 * rowMeans (ages[ ,c("HAGE", "LAGE")])
```

Split dataframes by location

```
agesMM = ages[ which (ages$grp == "MM"), ]
agesME = ages[ which (ages$grp == "ME"), ]
agesLT = ages[ which (ages$grp == "LT"), ]
```

Plot all regressions

Make plotting easier by using `lapply()` to generate plots, for which one needs a vector to loop over.

```
y_axis <- c("AMMN", "NITR", "POTA", "SLPH", "COND", "PHCC", "PHOS", "CARB",
               "PHHO")
```

Create a list of graphical objects for plotting on one page.

```
regr_listMM <- lapply (y_axis, plot_regrs, agesMM)
regr_listME <- lapply (y_axis, plot_regrs, agesME)
regr_listLT <- lapply (y_axis, plot_regrs, agesLT)
```

Plots can now be shown, and saved to the output directory. Objects are then discarded.

```
marrangeGrob (regr_listMM, nrow = 3, ncol = 3)
```

```
marrangeGrob (regr_listME, nrow = 3, ncol = 3)
```

```
marrangeGrob (regr_listLT, nrow = 3, ncol = 3)
```

Testing all regressions

Calculate all regressions, with variables from previous section, and store as list.

```
regressionsMM <- lapply (y_axis, function(x) {
    lm ( substitute (MNAGE ~ i, list(i = as.name(x))), data = agesMM)
    })

regressionsME <- lapply (y_axis, function(x) {
    lm ( substitute (MNAGE ~ i, list(i = as.name(x))), data = agesME)
    })

regressionsLT <- lapply (y_axis, function(x) {
    lm ( substitute (MNAGE ~ i, list(i = as.name(x))), data = agesLT)
    })
```

Return summary from list for more specific results

```
lapply (regressionsMM, summary )
```

```
## [[1]]
##
## Call:
## lm(formula = substitute(MNAGE ~ i, list(i = as.name(x))), data = agesMM)
##
## Residuals:
##      2.5.C     1.10.C     1.10.G
##  2.328e-10 -9.993e+05  9.993e+05
##
## Coefficients:
##             Estimate Std. Error t value Pr(>|t|)
## (Intercept) -1000750     999250  -1.002     0.50
## AMMN          494375     865376   0.571     0.67
##
## Residual standard error: 1413000 on 1 degrees of freedom
## Multiple R-squared:  0.2461, Adjusted R-squared:  -0.5079
## F-statistic: 0.3264 on 1 and 1 DF,  p-value: 0.6696
```

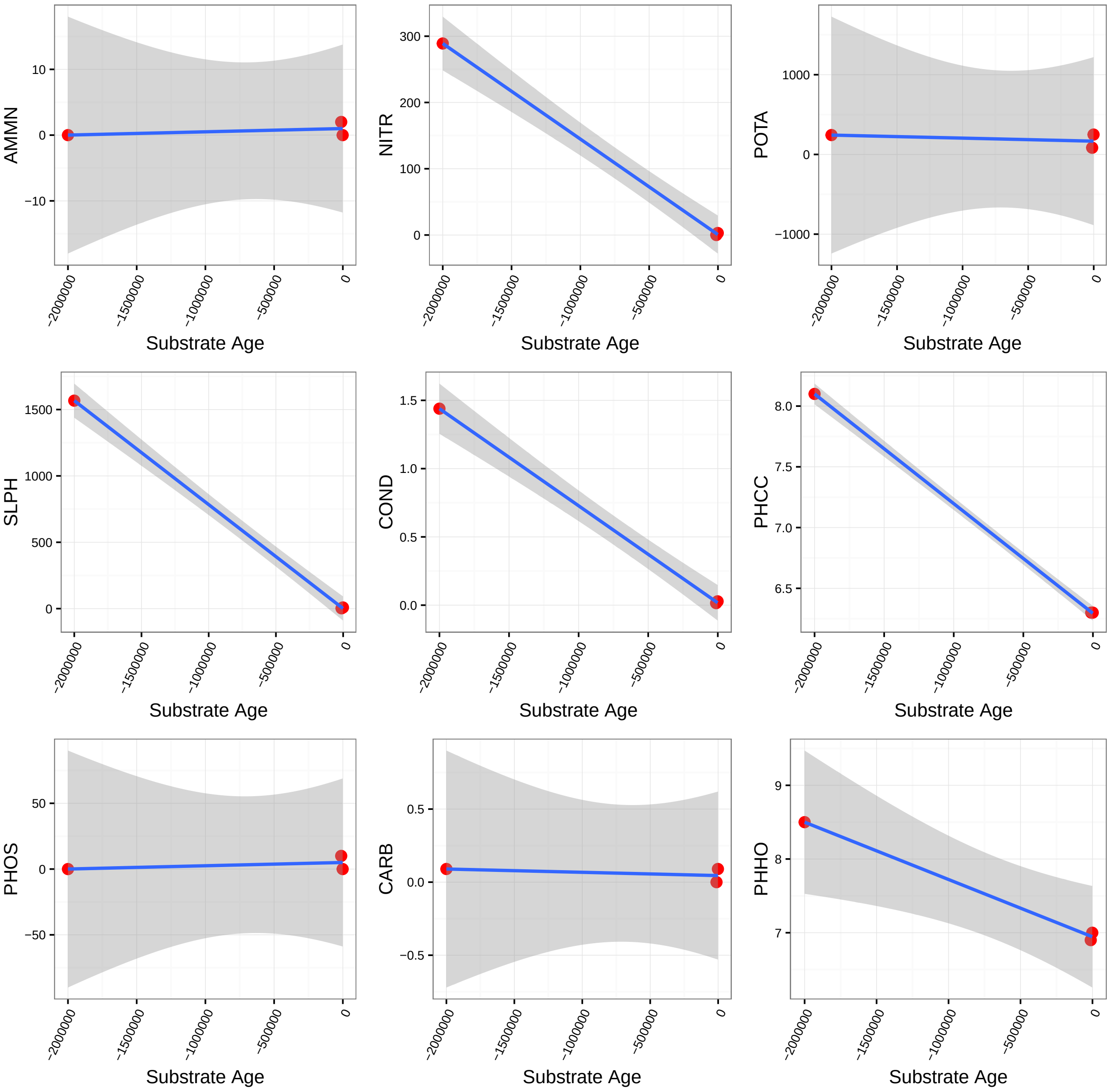

Figure 17: Mount Menzies: Regressions between terrrain age and soil geochemical measurements (Approx. code line 689).

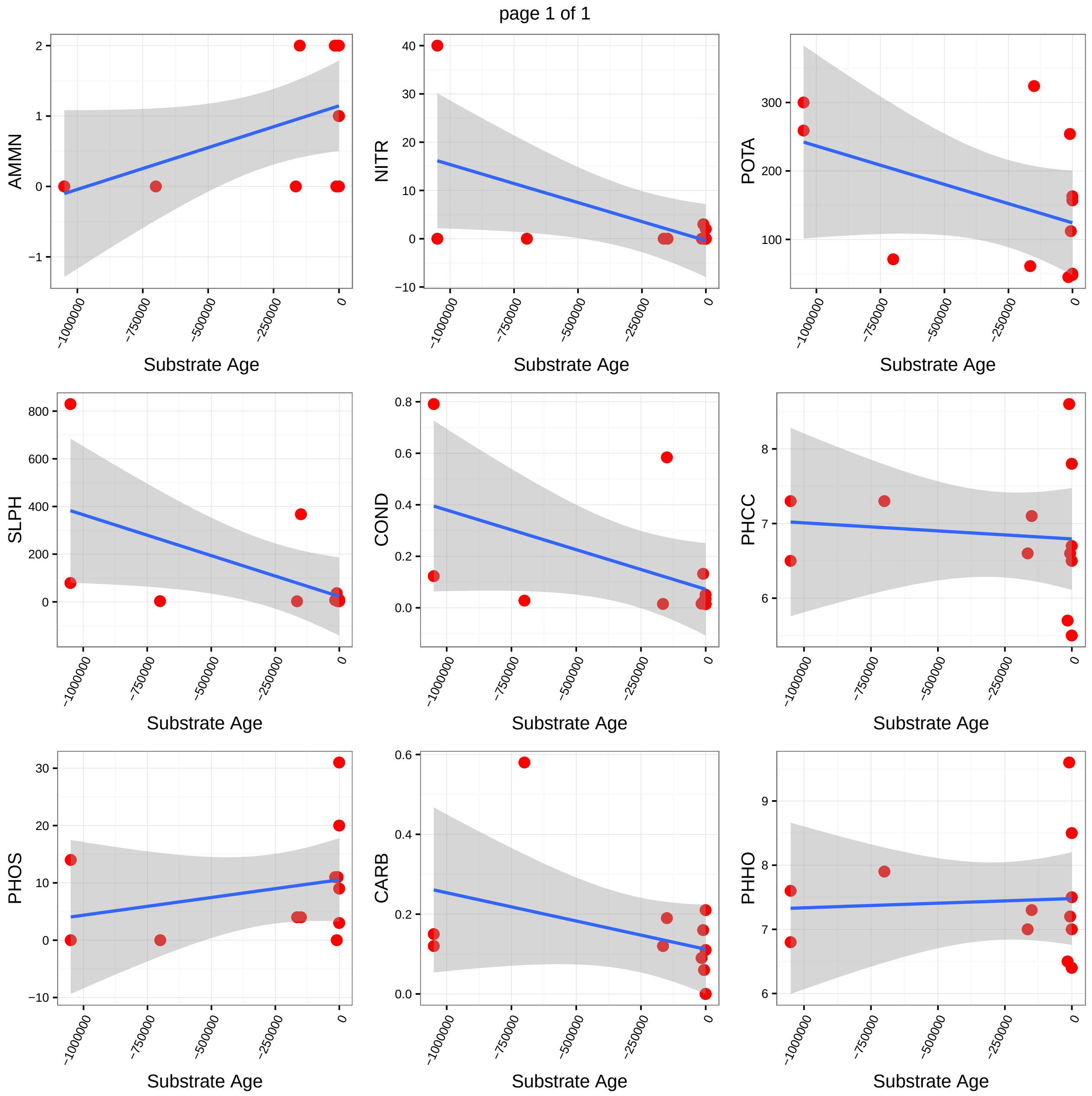

Figure 18: Mawson Escarpment: Regressions between terrain age and soil geochemical measurements (Approx. code line 692).

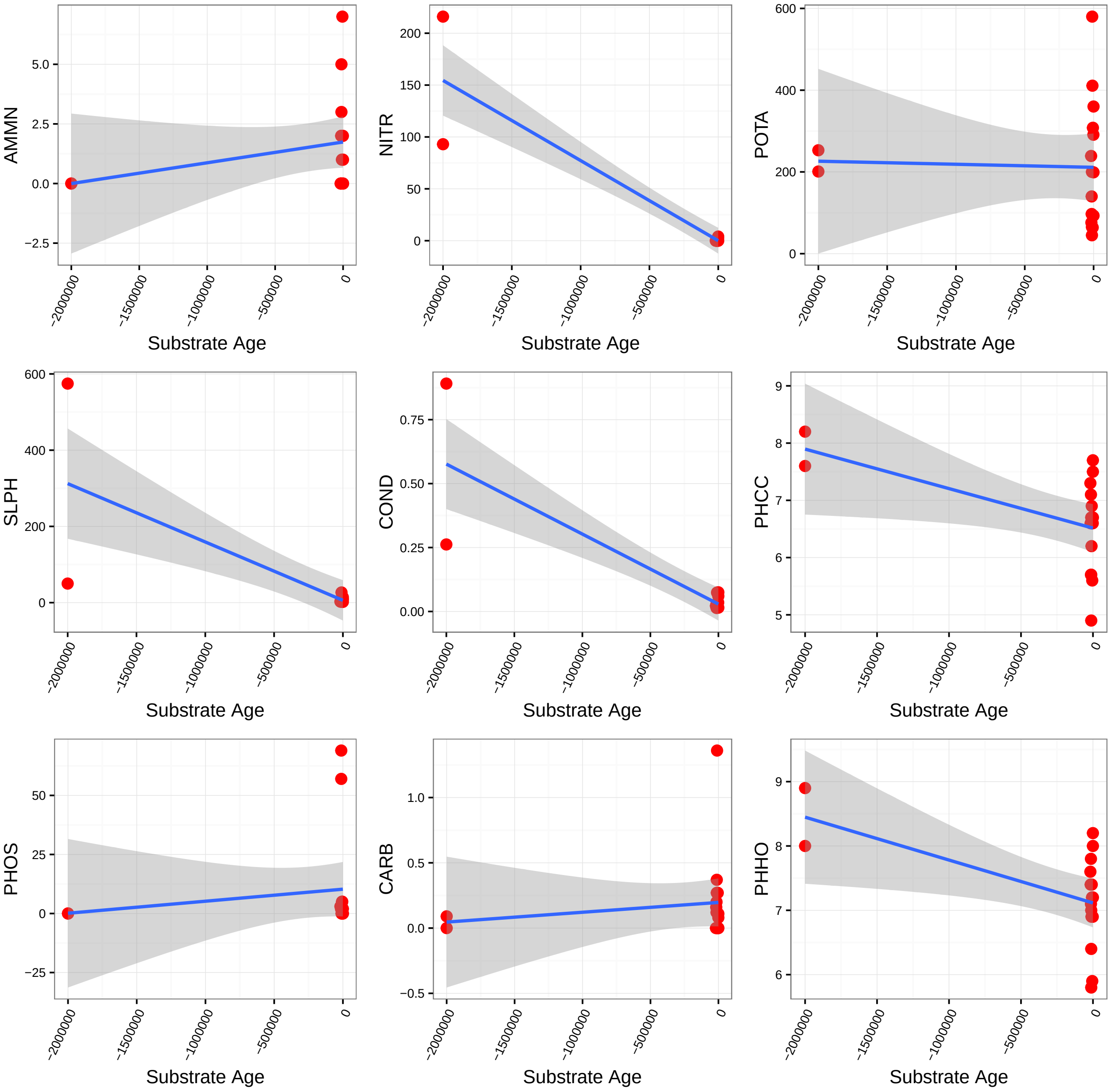

Figure 19: Lake Terrasovoje: Regressions between terrain age and soil geochemical measurements (Approx. code line 695).

```
##
##
## [[2]]
##
## Call:
## lm(formula = substitute(MNAGE ~ i, list(i = as.name(x))), data = agesMM)
##
## Residuals:
##    2.5.C   1.10.C   1.10.G
## -15566.6   -163.3  15729.9
##
## Coefficients:
##             Estimate Std. Error t value Pr(>|t|)
## (Intercept)  3566.64   15730.78   0.227  0.85806
## NITR        -6932.19      94.27 -73.533  0.00866 **
## ---
## Signif. codes:  0 '***' 0.001 '**' 0.01 '*' 0.05 '.' 0.1 ' ' 1
##
## Residual standard error: 22130 on 1 degrees of freedom
## Multiple R-squared:  0.9998, Adjusted R-squared:  0.9996
## F-statistic:  5407 on 1 and 1 DF,  p-value: 0.008657
##
##
## [[3]]
##
## Call:
## lm(formula = substitute(MNAGE ~ i, list(i = as.name(x))), data = agesMM)
##
## Residuals:
##    2.5.C   1.10.C   1.10.G
##    37649 -1035339   997690
##
## Coefficients:
##             Estimate Std. Error t value Pr(>|t|)
## (Intercept)   433754    2245863   0.193    0.879
## POTA           -5755      10868  -0.530    0.690
##
## Residual standard error: 1438000 on 1 degrees of freedom
## Multiple R-squared:  0.219,  Adjusted R-squared:  -0.562
## F-statistic: 0.2804 on 1 and 1 DF,  p-value: 0.69
##
##
## [[4]]
##
## Call:
## lm(formula = substitute(MNAGE ~ i, list(i = as.name(x))), data = agesMM)
##
## Residuals:
##    2.5.C   1.10.C   1.10.G
## -9125.97   -35.73  9161.70
##
## Coefficients:
##             Estimate Std. Error  t value Pr(>|t|)
## (Intercept)   445.31    9177.08    0.049  0.96913
```

```
## SLPH        -1276.67      10.14 -125.852  0.00506 **
## ---
## Signif. codes:  0 '***' 0.001 '**' 0.01 '*' 0.05 '.' 0.1 ' ' 1
##
## Residual standard error: 12930 on 1 degrees of freedom
## Multiple R-squared:  0.9999, Adjusted R-squared:  0.9999
## F-statistic: 1.584e+04 on 1 and 1 DF,  p-value: 0.005058
##
##
## [[5]]
##
## Call:
## lm(formula = substitute(MNAGE ~ i, list(i = as.name(x))), data = agesMM)
##
## Residuals:
##  2.5.C 1.10.C 1.10.G
## -14323   -132  14455
##
## Coefficients:
##             Estimate Std. Error t value Pr(>|t|)
## (Intercept)    23414      14611   1.602  0.35517
## COND        -1406033      17582 -79.969  0.00796 **
## ---
## Signif. codes:  0 '***' 0.001 '**' 0.01 '*' 0.05 '.' 0.1 ' ' 1
##
## Residual standard error: 20350 on 1 degrees of freedom
## Multiple R-squared:  0.9998, Adjusted R-squared:  0.9997
## F-statistic:  6395 on 1 and 1 DF,  p-value: 0.00796
##
##
## [[6]]
##
## Call:
## lm(formula = substitute(MNAGE ~ i, list(i = as.name(x))), data = agesMM)
##
## Residuals:
##      2.5.C     1.10.C     1.10.G
## -5.250e+03  2.728e-12  5.250e+03
##
## Coefficients:
##             Estimate Std. Error t value Pr(>|t|)
## (Intercept)  6969625      35120   198.5  0.00321 **
## PHCC        -1107361       5052  -219.2  0.00290 **
## ---
## Signif. codes:  0 '***' 0.001 '**' 0.01 '*' 0.05 '.' 0.1 ' ' 1
##
## Residual standard error: 7425 on 1 degrees of freedom
## Multiple R-squared:      1,  Adjusted R-squared:      1
## F-statistic: 4.805e+04 on 1 and 1 DF,  p-value: 0.002904
##
##
## [[7]]
##
## Call:
```

```
## lm(formula = substitute(MNAGE ~ i, list(i = as.name(x))), data = agesMM)
##
## Residuals:
##      2.5.C     1.10.C     1.10.G
##  2.328e-10 -9.993e+05  9.993e+05
##
## Coefficients:
##             Estimate Std. Error t value Pr(>|t|)
## (Intercept) -1000750     999250  -1.002     0.50
## PHOS           98875     173075   0.571     0.67
##
## Residual standard error: 1413000 on 1 degrees of freedom
## Multiple R-squared:  0.2461, Adjusted R-squared:  -0.5079
## F-statistic: 0.3264 on 1 and 1 DF,  p-value: 0.6696
##
##
## [[8]]
##
## Call:
## lm(formula = substitute(MNAGE ~ i, list(i = as.name(x))), data = agesMM)
##
## Residuals:
##   2.5.C  1.10.C  1.10.G
##       0 -999250  999250
##
## Coefficients:
##              Estimate Std. Error t value Pr(>|t|)
## (Intercept)    -12000    1413153  -0.008    0.995
## CARB        -10986111   19230575  -0.571    0.670
##
## Residual standard error: 1413000 on 1 degrees of freedom
## Multiple R-squared:  0.2461, Adjusted R-squared:  -0.5079
## F-statistic: 0.3264 on 1 and 1 DF,  p-value: 0.6696
##
##
## [[9]]
##
## Call:
## lm(formula = substitute(MNAGE ~ i, list(i = as.name(x))), data = agesMM)
##
## Residuals:
##  2.5.C 1.10.C 1.10.G
## -67095  -4473  71568
##
## Coefficients:
##             Estimate Std. Error t value Pr(>|t|)
## (Intercept)  8898405     581253   15.31   0.0415 *
## PHHO        -1281639      77475  -16.54   0.0384 *
## ---
## Signif. codes:  0 '***' 0.001 '**' 0.01 '*' 0.05 '.' 0.1 ' ' 1
##
## Residual standard error: 98200 on 1 degrees of freedom
## Multiple R-squared:  0.9964, Adjusted R-squared:  0.9927
## F-statistic: 273.7 on 1 and 1 DF,  p-value: 0.03844
```

```r
lapply (regressionsME, summary )
```

```
## [[1]]
##
## Call:
## lm(formula = substitute(MNAGE ~ i, list(i = as.name(x))), data = agesME)
##
## Residuals:
##     Min      1Q  Median      3Q     Max
## -591198 -182116  -15922  240468  458302
##
## Coefficients:
##             Estimate Std. Error t value Pr(>|t|)
## (Intercept)  -458802     146434  -3.133   0.0106 *
## AMMN          235612     119563   1.971   0.0771 .
## ---
## Signif. codes:  0 '***' 0.001 '**' 0.01 '*' 0.05 '.' 0.1 ' ' 1
##
## Residual standard error: 371700 on 10 degrees of freedom
## Multiple R-squared:  0.2797, Adjusted R-squared:  0.2077
## F-statistic: 3.883 on 1 and 10 DF,  p-value: 0.07707
##
##
## [[2]]
##
## Call:
## lm(formula = substitute(MNAGE ~ i, list(i = as.name(x))), data = agesME)
##
## Residuals:
##     Min      1Q  Median      3Q     Max
## -866009    6987  172741  183491  236765
##
## Coefficients:
##             Estimate Std. Error t value Pr(>|t|)
## (Intercept)  -183991     109409  -1.682   0.1235
## NITR          -20925       9437  -2.217   0.0509 .
## ---
## Signif. codes:  0 '***' 0.001 '**' 0.01 '*' 0.05 '.' 0.1 ' ' 1
##
## Residual standard error: 358600 on 10 degrees of freedom
## Multiple R-squared:  0.3296, Adjusted R-squared:  0.2626
## F-statistic: 4.917 on 1 and 10 DF,  p-value: 0.05092
##
##
## [[3]]
##
## Call:
## lm(formula = substitute(MNAGE ~ i, list(i = as.name(x))), data = agesME)
##
## Residuals:
##     Min      1Q  Median      3Q     Max
## -602305 -181678   77894  270458  428902
##
## Coefficients:
```

```
##             Estimate Std. Error t value Pr(>|t|)
## (Intercept)     7776     206128   0.038    0.971
## POTA           -1759       1121  -1.569    0.148
##
## Residual standard error: 392400 on 10 degrees of freedom
## Multiple R-squared:  0.1976, Adjusted R-squared:  0.1173
## F-statistic: 2.462 on 1 and 10 DF,  p-value: 0.1477
##
##
## [[4]]
##
## Call:
## lm(formula = substitute(MNAGE ~ i, list(i = as.name(x))), data = agesME)
##
## Residuals:
##     Min      1Q  Median      3Q     Max
## -819867  -30077  152811  158765  359437
##
## Coefficients:
##              Estimate Std. Error t value Pr(>|t|)
## (Intercept) -153501.1   114282.9  -1.343   0.2089
## SLPH           -968.8      434.3  -2.231   0.0498 *
## ---
## Signif. codes:  0 '***' 0.001 '**' 0.01 '*' 0.05 '.' 0.1 ' ' 1
##
## Residual standard error: 357900 on 10 degrees of freedom
## Multiple R-squared:  0.3323, Adjusted R-squared:  0.2655
## F-statistic: 4.977 on 1 and 10 DF,  p-value: 0.04977
##
##
## [[5]]
##
## Call:
## lm(formula = substitute(MNAGE ~ i, list(i = as.name(x))), data = agesME)
##
## Residuals:
##     Min      1Q  Median      3Q     Max
## -811604  -77905  149647  169574  463356
##
## Coefficients:
##             Estimate Std. Error t value Pr(>|t|)
## (Intercept)  -138353     128822  -1.074   0.3081
## COND         -813361     445025  -1.828   0.0975 .
## ---
## Signif. codes:  0 '***' 0.001 '**' 0.01 '*' 0.05 '.' 0.1 ' ' 1
##
## Residual standard error: 379200 on 10 degrees of freedom
## Multiple R-squared:  0.2504, Adjusted R-squared:  0.1754
## F-statistic:  3.34 on 1 and 10 DF,  p-value: 0.09755
##
##
## [[6]]
##
## Call:
```

```
## lm(formula = substitute(MNAGE ~ i, list(i = as.name(x))), data = agesME)
##
## Residuals:
##     Min      1Q  Median      3Q     Max
## -805691  -40181  189391  246402  343203
##
## Coefficients:
##             Estimate Std. Error t value Pr(>|t|)
## (Intercept)    92740    1064282   0.087    0.932
## PHCC          -51854     154282  -0.336    0.744
##
## Residual standard error: 435600 on 10 degrees of freedom
## Multiple R-squared:  0.01117,    Adjusted R-squared:  -0.08771
## F-statistic: 0.113 on 1 and 10 DF,  p-value: 0.7437
##
##
## [[7]]
##
## Call:
## lm(formula = substitute(MNAGE ~ i, list(i = as.name(x))), data = agesME)
##
## Residuals:
##     Min      1Q  Median      3Q     Max
## -849809  -88490  165184  238064  361681
##
## Coefficients:
##             Estimate Std. Error t value Pr(>|t|)
## (Intercept)  -371681     171351  -2.169   0.0553 .
## PHOS           12249      13543   0.904   0.3870
## ---
## Signif. codes:  0 '***' 0.001 '**' 0.01 '*' 0.05 '.' 0.1 ' ' 1
##
## Residual standard error: 421100 on 10 degrees of freedom
## Multiple R-squared:  0.07562,    Adjusted R-squared:  -0.01682
## F-statistic: 0.8181 on 1 and 10 DF,  p-value: 0.387
##
##
## [[8]]
##
## Call:
## lm(formula = substitute(MNAGE ~ i, list(i = as.name(x))), data = agesME)
##
## Residuals:
##     Min      1Q  Median      3Q     Max
## -819190   56845  128432  191557  327968
##
## Coefficients:
##             Estimate Std. Error t value Pr(>|t|)
## (Intercept)  -100599     167234  -0.602    0.561
## CARB        -1085092     805461  -1.347    0.208
##
## Residual standard error: 403000 on 10 degrees of freedom
## Multiple R-squared:  0.1536, Adjusted R-squared:  0.06897
## F-statistic: 1.815 on 1 and 10 DF,  p-value: 0.2077
```

```
##
##
## [[9]]
##
## Call:
## lm(formula = substitute(MNAGE ~ i, list(i = as.name(x))), data = agesME)
##
## Residuals:
##     Min      1Q  Median      3Q     Max
## -792469  -29548  207153  266536  294377
##
## Coefficients:
##             Estimate Std. Error t value Pr(>|t|)
## (Intercept)  -494060    1099298  -0.449    0.663
## PHHO           31122     146746   0.212    0.836
##
## Residual standard error: 437000 on 10 degrees of freedom
## Multiple R-squared:  0.004478,   Adjusted R-squared:  -0.09507
## F-statistic: 0.04498 on 1 and 10 DF,  p-value: 0.8363
```

```
lapply (regressionsLT, summary )
```

```
## [[1]]
##
## Call:
## lm(formula = substitute(MNAGE ~ i, list(i = as.name(x))), data = agesLT)
##
## Residuals:
##      Min       1Q   Median       3Q      Max
## -1607181    86943   195402   374819   392319
##
## Coefficients:
##             Estimate Std. Error t value Pr(>|t|)
## (Intercept)  -392820     202825  -1.937   0.0718 .
## AMMN           97959      82803   1.183   0.2552
## ---
## Signif. codes:  0 '***' 0.001 '**' 0.01 '*' 0.05 '.' 0.1 ' ' 1
##
## Residual standard error: 653200 on 15 degrees of freedom
## Multiple R-squared:  0.08534,    Adjusted R-squared:  0.02436
## F-statistic:   1.4 on 1 and 15 DF,  p-value: 0.2552
##
##
## [[2]]
##
## Call:
## lm(formula = substitute(MNAGE ~ i, list(i = as.name(x))), data = agesLT)
##
## Residuals:
##     Min      1Q  Median      3Q     Max
## -943873   23463   32463   38416  403393
##
## Coefficients:
##             Estimate Std. Error t value Pr(>|t|)
## (Intercept)   -37463      68828  -0.544    0.594
```

```
## NITR          -10953       1206  -9.079 1.75e-07 ***
## ---
## Signif. codes:  0 '***' 0.001 '**' 0.01 '*' 0.05 '.' 0.1 ' ' 1
##
## Residual standard error: 268000 on 15 degrees of freedom
## Multiple R-squared:  0.846,  Adjusted R-squared:  0.8358
## F-statistic: 82.43 on 1 and 15 DF,  p-value: 1.753e-07
##
##
## [[3]]
##
## Call:
## lm(formula = substitute(MNAGE ~ i, list(i = as.name(x))), data = agesLT)
##
## Residuals:
##      Min       1Q   Median       3Q      Max
## -1758872   208015   223862   252739   289965
##
## Coefficients:
##              Estimate Std. Error t value Pr(>|t|)
## (Intercept) -209924.3   300086.3  -0.700    0.495
## POTA           -155.2     1174.7  -0.132    0.897
##
## Residual standard error: 682600 on 15 degrees of freedom
## Multiple R-squared:  0.001163,   Adjusted R-squared:  -0.06543
## F-statistic: 0.01746 on 1 and 15 DF,  p-value: 0.8966
##
##
## [[4]]
##
## Call:
## lm(formula = substitute(MNAGE ~ i, list(i = as.name(x))), data = agesLT)
##
## Residuals:
##      Min       1Q   Median       3Q      Max
## -1732962    90774   101703   118393   174459
##
## Coefficients:
##             Estimate Std. Error t value Pr(>|t|)
## (Intercept) -89831.2   117747.0  -0.763 0.457344
## SLPH         -3544.1      839.9  -4.220 0.000742 ***
## ---
## Signif. codes:  0 '***' 0.001 '**' 0.01 '*' 0.05 '.' 0.1 ' ' 1
##
## Residual standard error: 461800 on 15 degrees of freedom
## Multiple R-squared:  0.5428, Adjusted R-squared:  0.5123
## F-statistic: 17.81 on 1 and 15 DF,  p-value: 0.0007424
##
##
## [[5]]
##
## Call:
## lm(formula = substitute(MNAGE ~ i, list(i = as.name(x))), data = agesLT)
##
```

```
## Residuals:
##      Min       1Q   Median       3Q      Max
## -1319218    30954    37856    82531   334334
##
## Coefficients:
##             Estimate Std. Error t value Pr(>|t|)
## (Intercept)     7979      96634   0.083    0.935
## COND        -2628858     423914  -6.201  1.7e-05 ***
## ---
## Signif. codes:  0 '***' 0.001 '**' 0.01 '*' 0.05 '.' 0.1 ' ' 1
##
## Residual standard error: 361800 on 15 degrees of freedom
## Multiple R-squared:  0.7194, Adjusted R-squared:  0.7007
## F-statistic: 38.46 on 1 and 15 DF,  p-value: 1.696e-05
##
##
## [[6]]
##
## Call:
## lm(formula = substitute(MNAGE ~ i, list(i = as.name(x))), data = agesLT)
##
## Residuals:
##      Min       1Q   Median       3Q      Max
## -1387069  -167016   203301   322240   652745
##
## Coefficients:
##             Estimate Std. Error t value Pr(>|t|)
## (Intercept)  2450860    1128815   2.171   0.0464 *
## PHCC         -403130     167607  -2.405   0.0295 *
## ---
## Signif. codes:  0 '***' 0.001 '**' 0.01 '*' 0.05 '.' 0.1 ' ' 1
##
## Residual standard error: 580200 on 15 degrees of freedom
## Multiple R-squared:  0.2783, Adjusted R-squared:  0.2302
## F-statistic: 5.785 on 1 and 15 DF,  p-value: 0.02952
##
##
## [[7]]
##
## Call:
## lm(formula = substitute(MNAGE ~ i, list(i = as.name(x))), data = agesLT)
##
## Residuals:
##      Min       1Q   Median       3Q      Max
## -1708905   257168   261168   279977   290595
##
## Coefficients:
##             Estimate Std. Error t value Pr(>|t|)
## (Intercept)  -291095     179580  -1.621    0.126
## PHOS            5309       8223   0.646    0.528
##
## Residual standard error: 673700 on 15 degrees of freedom
## Multiple R-squared:  0.02704,    Adjusted R-squared:  -0.03782
## F-statistic: 0.4169 on 1 and 15 DF,  p-value: 0.5283
```

```
##
##
## [[8]]
##
## Call:
## lm(formula = substitute(MNAGE ~ i, list(i = as.name(x))), data = agesLT)
##
## Residuals:
##      Min       1Q   Median       3Q      Max
## -1729653   202559   247049   286241   297741
##
## Coefficients:
##             Estimate Std. Error t value Pr(>|t|)
## (Intercept)  -298241     187944  -1.587    0.133
## CARB          309931     517887   0.598    0.558
##
## Residual standard error: 675000 on 15 degrees of freedom
## Multiple R-squared:  0.02332,    Adjusted R-squared:  -0.04179
## F-statistic: 0.3581 on 1 and 15 DF,  p-value: 0.5585
##
##
## [[9]]
##
## Call:
## lm(formula = substitute(MNAGE ~ i, list(i = as.name(x))), data = agesLT)
##
## Residuals:
##      Min       1Q   Median       3Q      Max
## -1425761  -170257   148210   291053   665301
##
## Coefficients:
##             Estimate Std. Error t value Pr(>|t|)
## (Intercept)  3088239    1305048   2.366   0.0318 *
## PHHO         -457810     178345  -2.567   0.0215 *
## ---
## Signif. codes:  0 '***' 0.001 '**' 0.01 '*' 0.05 '.' 0.1 ' ' 1
##
## Residual standard error: 569300 on 15 degrees of freedom
## Multiple R-squared:  0.3052, Adjusted R-squared:  0.2589
## F-statistic: 6.589 on 1 and 15 DF,  p-value: 0.02146
```

Write data to disk

Saved are object created by this script as well as command history and work-space image.

```
save.image (path_workspace_b) # work-space
```

Session info

The code and output in this document were tested and generated in the following computing environment:

```
## R version 3.3.1 (2016-06-21)
```

```
## Platform: x86_64-apple-darwin13.4.0 (64-bit)
## Running under: OS X 10.11.6 (El Capitan)
##
## locale:
## [1] en_AU.UTF-8/en_AU.UTF-8/en_AU.UTF-8/C/en_AU.UTF-8/en_AU.UTF-8
##
## attached base packages:
## [1] grid      stats     graphics  grDevices utils     datasets  methods
## [8] base
##
## other attached packages:
##  [1] caret_6.0-71      rgr_1.1.13        fastICA_1.2-0
##  [4] MASS_7.3-45       outliers_0.14     maps_3.1.1
##  [7] phylogeo_0.99.6.3 dplyr_0.5.0       vegan_2.4-1
## [10] lattice_0.20-34   permute_0.9-4     GGally_1.2.0
## [13] gridExtra_2.2.1   ggcorrplot_0.1.1  corrplot_0.77
## [16] ggbiplot_0.55     scales_0.4.0      plyr_1.8.4
## [19] ggplot2_2.1.0     phyloseq_1.16.2
##
## loaded via a namespace (and not attached):
##  [1] Biobase_2.32.0      jsonlite_1.1        splines_3.3.1
##  [4] foreach_1.4.3       Formula_1.2-1       assertthat_0.1
##  [7] sp_1.2-3            stats4_3.3.1        latticeExtra_0.6-28
## [10] yaml_2.1.13         quantreg_5.29       chron_2.3-47
## [13] digest_0.6.10       RColorBrewer_1.1-2  XVector_0.12.1
## [16] minqa_1.2.4         colorspace_1.2-7    cowplot_0.6.3
## [19] htmltools_0.3.5     Matrix_1.2-7.1      SparseM_1.72
## [22] zlibbioc_1.18.0     MatrixModels_0.4-1  lme4_1.1-12
## [25] tibble_1.2          mgcv_1.8-15         IRanges_2.6.1
## [28] car_2.1-3           nnet_7.3-12         BiocGenerics_0.18.0
## [31] pbkrtest_0.4-6      survival_2.39-5     magrittr_1.5
## [34] evaluate_0.10       nlme_3.1-128        foreign_0.8-67
## [37] tools_3.3.1         data.table_1.9.6    formatR_1.4
## [40] stringr_1.1.0       S4Vectors_0.10.3    munsell_0.4.3
## [43] cluster_2.0.5       Biostrings_2.40.2   ade4_1.7-4
## [46] nloptr_1.0.4        rhdf5_2.16.0        iterators_1.0.8
## [49] biomformat_1.0.2    htmlwidgets_0.7     igraph_1.0.1
## [52] labeling_0.3        rmarkdown_1.0       gtable_0.2.0
## [55] codetools_0.2-15    multtest_2.28.0     DBI_0.5-1
## [58] reshape_0.8.5       reshape2_1.4.1      R6_2.2.0
## [61] knitr_1.14          Hmisc_3.17-4        ape_3.5
## [64] stringi_1.1.2       parallel_3.3.1      Rcpp_0.12.7
## [67] mapproj_1.2-4       rpart_4.1-10        acepack_1.3-3.3
## [70] leaflet_1.0.1
```

References

McMurdie, Paul J, and Susan Holmes. 2013. "Phyloseq: An R Package for reproducible interactive analysis and graphics of microbiome census data." *PLoS ONE* 8 (4): e61217.

Paulson, Joseph N, O Colin Stine, Héctor Corrada Bravo, and Mihai Pop. 2013. "Differential abundance

analysis for microbial marker-gene surveys." *Nature Methods* 10 (12): 1200–1202. doi:10.1038/nmeth.2658.

Ranganathan, Yuvaraj, and Renee M Borges. 2011. "To transform or not to transform." *Plant Signaling & Behavior* 6 (1): 113–16. doi:10.4161/psb.6.1.14191.
